# Analytical sameness methodology for the evaluation of structural, physicochemical, and biological characteristics of Armlupeg: A pegfilgrastim biosimilar case study

**DOI:** 10.1371/journal.pone.0289745

**Published:** 2023-08-09

**Authors:** Arati Deshmukh, Rishank Goyal, Kalyana Sundaram, Kaustubh Dange, Tejshri Lakhote, Sanjay Niranjan, Jennifer Bharucha, Ashok Mishra, Bhavesh Vats, Sanjay Tiwari

**Affiliations:** Research and Development, Lupin Limited (Biotechnology Division), Pune, Maharashtra, India; University of Colorado Anschutz Medical Campus, UNITED STATES

## Abstract

Pegfilgrastim is administered as an adjunct to chemotherapy to reduce the incidence of febrile neutropenia and associated infectious complications. Lupin’s Pegfilgrastim is a proposed biosimilar to the U.S.-referenced Neulasta®. Demonstration of biosimilarity requires extensive physicochemical and functional characterization of the biosimilar, and demonstration of analytical similarity to the reference product, in addition to clinical studies. This work is a case study for demonstrating the analytical similarity of Armlupeg (Lupin’s Pegfilgrastim) to Neulasta® with respect to structural and physicochemical attributes using several robust, orthogonal, and state-of-the-art techniques including high-end liquid chromatography, mass spectrometry, and spectroscopy techniques; circular dichroism; differential scanning calorimetry; nuclear magnetic resonance; analytical ultracentrifugation; and micro-flow imaging. Functional similarity was demonstrated using an *in vitro* cell proliferation assay to measure relative potency and surface plasmon resonance to measure receptor binding kinetics. Furthermore, comparative forced-degradation studies were performed to study the degradation of the products under stress conditions. The product attributes were ranked based on a critical quality attributes risk score according to their potential clinical impact. Based on criticality, all analyses were statistically evaluated to conclude analytical similarity. Lupin’s Pegfilgrastim was comparable to Neulasta® as demonstrated via structural, functional, and purity analyses. Lupin’s Pegfilgrastim complied with the quality and statistical ranges established using Neulasta®. Both products follow the same degradation pathways under stress conditions as observed in the forced-degradation studies. No new impurity or degradation product was observed in Lupin’s Pegfilgrastim. These data conclusively demonstrate the analytical similarity of Lupin’s Pegfilgrastim and Neulasta®.

## Introduction

Pegfilgrastim, a pegylated recombinant human granulocyte colony-stimulating factor (G-CSF), is administered to cancer patients to decrease the incidence of infection, as manifested by febrile neutropenia in patients with non-myeloid malignancies receiving myelosuppressive chemotherapy [1, 2]. Pegfilgrastim binds to the G-CSF receptor and stimulates the proliferation, differentiation, and activation of neutrophils [3]. It has a 20 kDa monomethoxy polyethylene glycol (PEG) molecule covalently bound to the N-terminal methionine residue of filgrastim, which is a 175 amino acid protein having four antiparallel helices with a molecular weight of 18.8 kDa [1, 4, 5].

A biosimilar is obtained from a biological source different from that of a biologic [6]. Biologics and biosimilars can be heterogeneous in structure, which can lead to variability in physicochemical characteristics including non-critical as well as critical quality attributes (CQA) [7].

First developed by Amgen, pegfilgrastim was approved by the U.S. Food and Drug Administration (FDA) in 2002 and marketed as Neulasta® [8]. Armlupeg (Lupin’s Pegfilgrastim) was developed as a biosimilar to the reference, U.S.-licensed Neulasta®. A phase I study in healthy volunteers has shown the pharmacokinetic and pharmacodynamic equivalence of Lupin’s Pegfilgrastim and Neulasta® (manuscript submitted). Furthermore, a phase III clinical trial has also demonstrated the therapeutic equivalence of Lupin’s Pegfilgrastim and the reference pegfilgrastim [9].

Though therapeutic, pharmacokinetic, and pharmacodynamic similarity are clinically important characteristics of a biosimilar, analytical similarity studies are significant for biosimilarity assessment [10–12]. Hence, a proposed biosimilar should demonstrate totality of evidence for similarity with the reference biologic in terms of quality, safety, and efficacy for regulatory approval [13, 14].

The aim of this study was to demonstrate the analytical similarity of Lupin’s Pegfilgrastim to Neulasta® with respect to physicochemical, structural, and functional attributes using a scientifically robust orthogonal methodology. We have focused on identifying differences in quality attributes between Lupin’s Pegfilgrastim and Neulasta® and extensively characterizing the two products using state-of-the-art techniques to statistically evaluate their analytical similarity. We also performed comparative forced-degradation studies to understand the differences in degradation pathways of the two products under stress conditions and the results have been used to demonstrate analytical similarity.

## Materials

For the analytical similarity assessment, the following lots were used: 1 to 12 lots of U.S.-licensed Neulasta® (Amgen), 7- to 35-months old, expiry ranging from May 2019 to January 2023; and 1 to 18 lots of Armlupeg (Lupin Limited, addressed as Lupin’s Pegfilgrastim), 1- to 33-months old, expiry ranging from February 2019 to September 2023. The internal primary reference standard, IPRS/004/20/001, also referred to as IPRS, was used either as a reference and/or system suitability assessment, as applicable.

All chemicals and products (S1 Appendix) were stored and handled according to manufacturer’s instructions.

## Methods

All methods were developed using appropriate positive and negative controls, wherever applicable (S2 Appendix).

### Western blot

Pegfilgrastim samples (1 μg) were resolved by sodium dodecyl sulfate polyacrylamide gel electrophoresis (SDS-PAGE) under reducing conditions on a 12% gel. The gel was run at 100 V and blotted onto nitrocellulose membrane (Biorad) using Biorad Trans-Blot Turbo Transfer system at 25 V for 7 min. Non-specific sites of the membrane were blocked using bovine serum albumin solution in phosphate buffered saline. Western blotting was done using mouse anti-filgrastim (MAB214, R&D systems, 1:1000) and rabbit anti-PEG (ab190652, Abcam, 1:2000) primary antibodies; and goat anti-mouse IgG (1706461, Biorad, 1:2000) and donkey anti-rabbit IgG (ab97061, Abcam, 1:1000) secondary antibodies for detection. The blot was developed with 5-bromo-4-chloro-3-indolyl phosphate (BCIP)/nitro blue tetrazolium (NBT) liquid substrate system (B1911, Sigma). The blots were imaged using a gel documentation system (Biorad).

### Peptide mapping by reverse phase high performance liquid chromatography (RP-HPLC)

Pegfilgrastim samples (80 μg) were enzymatically digested with endoproteinase Glu-C (Roche) under denaturing and reducing conditions using urea, dithiothreitol and methylamine for 18 h at 25°C with the enzyme:protein ratio of 1:40. The enzymatic reaction was quenched by acidification with 0.2% trifluoroacetic acid. The endoproteinase Glu-C digested samples were injected into a Waters XBridge Protein BEH C4 column (300 Å, 2.1 × 250 mm), and the peptides were separated by applying a linear gradient from 2 to 60% of mobile phase B (0.1% trifluoroacetic acid in 90% acetonitrile) and mobile phase A (0.1% trifluoroacetic acid in purified water) over 75 min and detected at 214 nm keeping the column temperature at 42°C with a flow rate of 0.2 mL/min.

For non-reduced peptide mapping, pegfilgrastim samples were denatured, and enzymatically digested with endoproteinase Glu-C for 20 h at 37°C with the enzyme:protein ratio of 1:10. The enzymatic reaction was quenched with 8 M guanidine hydrochloride. The endoproteinase Glu-C digested samples were injected into a YMC-Pack ODS-AQ column (20 nm, 100 × 2 mm column), and the peptides were separated by applying a linear gradient from 3 to 90% of mobile phase B (0.05% trifluoroacetic acid in 95% acetonitrile) and mobile phase A (0.05% trifluoroacetic acid in 5% acetonitrile) over 40 min and detected at 215 nm keeping the column temperature at 60°C with a flow rate of 0.2 mL/min.

For reduced and non-reduced peptide mapping, the HPLC was performed using Agilent 1260 Infinity LC System, and the peak distribution was obtained using Chromeleon^TM^ software version 6.8.

### Amino acid composition by reverse phase UPLC (RP-UPLC)

Amino acid composition was estimated using AccQ Tag Ultra Derivatization kit (Waters). Pegfilgrastim samples (200 μg) were hydrolyzed at 110°C for 24 h in vacuum hydrolysis tubes. After hydrolysis, samples were labeled with the dye 6-aminoquinolyl-N-hydroxysuccinimidyl carbamate (Waters) by incubating at 55°C for 10 min. The labeled samples were injected into a Waters Acc Q Tag C18 column (130 Å, 2.1 × 100 mm). The chromatographic column was equilibrated with 10% of solvent A (proprietary of Waters) and 90% of solvent B (proprietary of Waters). The peaks were separated using Waters ACQUITY UPLC H-Class System by applying a combination of a gradient of solvent A (10% to 4% in 8.5 min), solvent B (0% to 80% in 5.2 mins, 80% to 15.6 in 1.6 min), solvent C (90.1% to 36.3% in 8.3 mins) and solvent D (0% to 59.7% in 8.6 min) and detected at 260 nm keeping the column temperature at 43°C with a flow rate of 0.7 mL/min. Water was used as solvent C and solvent B was used as solvent D.

The amino acids eluted between 2 to 9 min. The mole % of each amino acid was calculated based on the calibration curve generated for respective amino acid standards.

### Liquid chromatography mass spectrometry (LC-MS) and tandem MS (MS/MS)

For amino acid sequence analysis, Pegfilgrastim samples were denatured with 8 M guanidine hydrochloride, reduced with 1M dithiothreitol, incubated at 37°C for 1 h, and alkylated in the dark with 0.5 M iodoacetamide for 30 minutes. The samples were then digested with proteases Glu-C (Promega) and Chymotrypsin (Promega) for 3.5 h at 37°C with the enzyme:protein ratio of 1:40. The digestion reaction was terminated with 0.9% formic acid. The analysis was performed using Thermo Scientific Vanquish UHPLC system connected to a Thermo Orbitrap Elite Mass Spectrometer. The digested peptides were resolved on a reverse phase Waters Acquity BEH C18 column (135 Å, 2.1 X 100 mm) by applying a linear gradient from 0.5 to 50% of solvent B (0.1% formic acid in 80% acetonitrile) and solvent A (0.1% formic acid in purified water) over 120 min and detected at 214 nm keeping the column temperature at 40°C with a flow rate of 0.15 mL/min. The eluted peptides were transferred online to the connected mass spectrometer for precursor m/z determination followed by fragment m/z determination. The total ion chromatogram (TIC) overlays were compared to indicate similarity in the peptide mapping profiles between test samples.

For N- and C-terminal heterogeneity, pegfilgrastim samples were processed and analyzed as described above except they were digested with Glu-C (for 3 h at 37°C with the enzyme:protein ratio of 1:40).

The LC-MS and MS/MS data were processed to determine the amino acid sequence and its coverage using Protein Metrics software while the oxidized peptides were processed using Xcalibur software.

For non-reduced peptide mapping, pegfilgrastim samples were denatured and alkylated as described above. The samples were then digested with proteases–Glu-C and Chymotrypsin for 2 h at 37°C with the enzyme:protein ratio of 1:40. The LC-MS was done as described above.

The LC-MS and MS/MS data were processed to determine the expected disulfide-bonded peptides and free cysteines, if any, present in the samples using Protein Metrics software.

### Electrospray ionization time-of-flight (ESI-TOF) MS

The intact mass was determined using an electrospray ionization time-of-flight (ESI-TOF) mass spectrometer (Waters Xevo G2-XS Q-TOF) coupled to Waters H class Bio UPLC. Pegfilgrastim samples were diluted to 0.5 mg/mL, and 10 μL of the diluted samples (5 μg) were injected into a Phenomenex Aries Widepore reverse phase C4 (200 Å, 2.1 × 150 mm) column. The peaks were separated by applying a linear gradient from 0.5 to 25% for 8 min and 25 to 90% for 2 min of solvent B (0.1% formic acid in 100% acetonitrile) and solvent A (0.1% formic acid in purified water) and detected at 214 nm keeping the column temperature at 60°C with a flow rate of 0.3 mL/min. Post-column infusion of tri-ethylamine was performed to reduce the charge complexity to yield a simplified spectrum in the online connected mass spectrometer. The ESI mass spectrum of TOF-MS data was deconvoluted using the protein deconvolution software Unify to obtain an intact protein mass spectrum. These data were used for determining the polydispersity index (PDI) based on the molecular weight and intensity of the polydisperse mass peaks of pegfilgrastim.

### Polydispersity analysis

The Eqs (1 to 3) were used for the dispersity analysis:

MN=Σ(MiNi)/ΣNi (1)

$$MW=\Sigma \;(Mi^{2}Ni)/\Sigma \;(MiNi)$$ (2)

PDI=MW/MN (3)

where MW is a weight-average molecular mass, MN is a number-average molecular mass, Mi is the deconvoluted mass for the i-meric PEG in the protein conjugate, Ni is the measured intensity for this mass, PDI is the dispersity index.

### One cycle Edman degradation

One cycle Edman degradation and further analyses were outsourced to BioPharmaSpec Inc. (USA).

Pegfilgrastim samples, 2 mg each, were dried in a vacuum evaporator and reconstituted in 0.5 mL 10% acetic acid/NaOH buffer (pH 4.0). To the reconstituted samples, 10 μL of 100% phenyl isothiocyanate (PITC) was added and incubated at 45°C for 30 min. Post incubation, the samples were flash frozen in liquid nitrogen and lyophilized. The lyophilized samples were reconstituted in 50% trifluoroacetic acid/50% water to achieve a final protein concentration of 2 μg/μL. The resulting samples were incubated for 10 min at room temperature, and a volume containing 167 μg of each sample was injected into a Waters BEH C8 column (130 Å, 3 mm × 50 mm) connected to a Waters Acquity UPLC. The separation was performed by applying the following acetonitrile gradient: 0 to 2 min, 10%; 2 to 22min, 45 to 55%; 22 to 23 min, 55 to 90%; 23 to 27 min, 90%; 27 to 32 min, 10% acetonitrile. The peaks were detected by a UV diode array detector (DAD) at wavelengths ranging from 210 to 400 nm and analyzed by online ESI-MS^e^ (Waters Xevo G2-XS Q-TOF Mass Spectrometer).

Protein (3.6 to 4.8 min) and PEG (16 to 23 min) fractions were separately collected, and volumes of the pooled fractions were reduced in a vacuum concentrator to obtain ~1 mg/mL for further analyses. The peptide mapping analysis of the collected protein fractions, intact molecular weight of PEG fractions, and depegylated protein intact mass (after one step Edman degradation) were performed at BioPharmaSpec Inc. (USA)

#### N-terminal pegylation analysis by peptide mapping LC-MS

The protein fractions purified and dried after one step of Edman degradation were reconstituted in 50 mM ammonium bicarbonate buffer (pH 8.4) and digested with Glu-C for 18 h at 37°C with the enzyme:protein ratio of 1:10,. Samples were further reductively alkylated with iodoacetic acid and analyzed for N-terminal pegylation by LC-MS. Peptide map analysis involved a manual fitting of the observed m/z values to theoretical peptide masses generated by Waters BioLynx Protein/Peptide Editor program from the MassLynx 4.1 software package based on the protein sequence. The LC-MS analysis was performed with a mass tolerance of 20 ppm.

#### Released PEG LC-MS analysis

PEG-Met fractions collected after one cycle of manual Edman degradation were lyophilized and reconstituted in 50% methanol/50% LC-MS grade water to a concentration of ~10 mg/mL. Buffer composition and sample concentrations were previously optimized for the ESI-MS analysis using a direct infusion to Waters Xevo G2-XS Q-TOF mass spectrometer calibrated in the 500 to 8000 m/z range. TIC was recorded for 1 min at 20 μL/min. Raw mass spectra were accumulated for 1 min infusion and deconvoluted to generate intact mass spectra after processing with MaxEnt1 program from the Waters MassLynx4.1 software package. The peak lists centered at about 22000 Da and covering 3000 Da were imported to Excel worksheets, and low-intensity peaks (at the noise level) were filtered out to produce a population of ~70 mass (+44 Da) isoforms considering the pseudo-molecular PEG-Met ions were charged with [DEMA + H+] adduct, thus including a respective mass of the adduct (+87 Da x charge state). The same parameters were used for processing the raw mass spectral data from both samples (MassLynx software): raw MS range, 2400 to 6200 m/z; MaxEnt1 deconvolution method range, 10,000 to 100,000 Da; resolution, 0.5 Da/channel; uniform Gaussian model [0.75 Da width at half height]; minimum intensity ratios, 33%; number of iterations, 15; center method, median [minimum peak width at half height, 2 channels, 90% of maximum]).

#### N-terminal pegylation analysis by released intact protein LC-MS

The protein fractions (des-PEG-Met Filgrastim) purified after one cycle of Edman degradation were analyzed for N-terminal pegylation by LC-MS of the released intact protein. Raw mass spectra of the peaks analyzed were deconvoluted to obtain intact mass after processing with MaxEnt1 program from the Waters MassLynx4.1 software package. The general mass tolerance for ESI-MS intact mass analysis was 0.01% or 100 ppm.

### Circular dichroism (CD)

Pegfilgrastim samples were diluted with purified water to a concentration of 0.1 mg/mL for far UV CD and 1 mg/mL for near UV CD. The respective buffers were diluted to the same fold as the pegfilgrastim samples. The baseline was recorded before sample run for blank subtraction. Samples were scanned using JASCO J-1500 CD spectrometer from 260 to 190 nm at a scan speed of 20 nm/min for far UV CD and from 350 to 250 nm at a scan speed of 20 nm/min for near UV CD. Scans were recorded at 20°C. Raw data were processed, and the secondary structure was predicted by CDNN deconvolution software.

### Fourier transform infrared spectroscopy (FTIR)

FTIR spectra were collected on a PerkinElmer Spectrum Two^TM^ FT-IR spectrometer equipped with a diamond attenuated total reflection (ATR) attachment. FTIR spectra of the pegfilgrastim samples (10 mg/mL) were acquired from 1750 to 1450 cm^-1^ at 4 cm^-1^ resolution with a data average of 128 scans for samples and buffer. Residual moisture peaks were subtracted from the spectrum of each sample followed by blank subtraction.

### Intrinsic fluorescence spectroscopy

Pegfilgrastim samples were diluted to a concentration of 0.5 mg/mL and the formulation buffer was diluted 20-fold in purified water. Samples were excited at 280 nm and 295 nm and the emission spectra were recorded using JASCO FP-8300 spectrofluorometer.

### Second derivative UV spectroscopy

Pegfilgrastim samples were diluted to 1 mg/mL and 0.5 mg/mL in purified water and samples were transferred to a quartz microcell with a path length of 10 mm. The spectral scan was performed (Agilent Cary 60 UV-visible spectrophotometer with Cary WinUV software) from 200 to 400 nm with intervals of 1 nm and second derivative spectra were generated. From the second derivative spectra, the a/b ratio was calculated as the difference in intensity of the 288±2 nm maximum and the 284±2 nm minimum peaks divided by the difference in intensity between the 296±2 nm maximum and the 291±2 nm minimum.

### Differential scanning calorimetry

Pegfilgrastim samples were diluted to 1 mg/mL in formulation buffer for differential scanning calorimetry (TA Instruments Nano DSC with DSC Run 4.6.0 software). All samples including formulation buffer and pegfilgrastim samples were degassed prior to analysis. First, a buffer scan was performed from 25 to 100°C at a heating rate of 1°C/min with formulation buffer in the reference and sample cell. After the first scan, the buffer was reheated from 25 to 100°C. Then the formulation buffer in the sample cell was replaced by 1 mg/mL pegfilgrastim solution. A temperature scan was performed from 25 to 100°C at a heating rate of 1°C/min. Using NanoAnalyze 3.11.0 software, the buffer scan was subtracted from the pegfilgrastim scan and the resultant thermogram was curve fitted to obtain the melting temperature of pegfilgrastim.

### Nuclear magnetic resonance (NMR)

The NMR analysis was outsourced to BioPharmaSpec Inc. (USA).

Pegfilgrastim samples were analyzed at a protein concentration of 257 μM. The NMR sample consisted of 540 μL of pegfilgrastim sample in the formulation buffer supplemented with 10% D_2_O made up to the final volume of 0.6 mL to establish a frequency lock signal. The 1D and 2D correlation NMR spectra were recorded at 303 K on a Bruker DRX800 equipped with a triple-resonance cryoprobe equipped with three channels (^1^H, ^13^C, and ^15^N).

### Cell proliferation assay using M-NFS-60 cell line

M-NFS-60 cells (ATCC, CRL-1838) were seeded in 96-well tissue culture plates (Corning, CLS3603) at a density of 5,000 cells/well and incubated at 37°C. Pegfilgrastim, at a concentration range of 0.5 to 3000 pg/mL, was added and the cells were further incubated for 44±2 h. Cell proliferation due to pegfilgrastim was measured by adding Cell Titer Glo detection reagent (Promega, G7571) to the assay plate and incubating at room temperature for 30 to 60 min with 15 min of intermittent shaking followed by 15 to 45 min without shaking. The luminescence signal was measured using a microplate reader (Spectramax M5, Molecular device). Relative potency was determined (using PLA 3.0 statistical software) by comparing the response curve of the test sample to that of the reference standard. For analytical similarity, the relative potency of each batch of Neulasta® and Lupin’s Pegfilgrastim was calculated from three independent determinants.

### Surface plasmon resonance (SPR)

The interaction of pegfilgrastim with G-CSF receptor was studied using SPR (BIAcore T200). The G-CSF receptor was immobilized on the flow cell surface 2 or 4 of the CM5 sensor chip (matrix of carboxymethylated dextran covalently attached to a gold surface) at 2000 RU through amine coupling. Surface 1 or 3 was used as the reference surface after blank immobilization. Seven concentrations (ranging from 0.08 to 5 nM) of pegfilgrastim were injected and a real-time response was measured. The change in refractive index was recorded as a sensorgram plotted as resonance units (RU) versus time. The binding affinity and kinetic data of pegfilgrastim binding to G-CSF receptor were calculated after fitting the generated sensorgram (plot of SPR response vs time) using BIA evaluation software with a 1:1 binding model. The binding kinetics and affinity data analysis were performed in triplicates for each batch and the corresponding arithmetic mean was calculated. The kinetics dissociation constant (K_D, Kinetics_) of pegfilgrastim-G-CSF receptor binding was used for statistical analysis.

### Size exclusion chromatography (SE-HPLC)

Pegfilgrastim samples were diluted to 1 mg/mL and 10 μL of diluted samples were injected into a TOSOH TSK gel G3000 SWXL silica column (25 nm, 7.8 × 300 mm) connected to Agilent 1260 Infinity LC System. The size variants were separated using 100 mM phosphate buffer (pH 6.4) with 10% ethanol and detected at 215 nm keeping the column temperature at 30°C with a flow rate of 0.5 mL/min. The peak distribution was obtained using Chromeleon^TM^ software version 6.8.

### Analytical ultracentrifugation (AUC)

AUC analysis was outsourced to KBI Biopharma (USA). Immediately prior to analysis, all the pegfilgrastim samples were diluted to 0.6 mg/mL. Sedimentation velocity AUC analysis was performed at 20°C and the data were recorded at 280 nm (Beckman Coulter ProteomeLab XL-I). Initial sedimentation velocity scans were undertaken at 3,000 rpm (using absorbance maximum at 280 nm) to check for the presence of heavy aggregates and confirm proper loading. The analysis was done at a final rotor speed of 60,000 rpm, and a total of 60 scans at every 4-min interval were recorded for ~4 h for each sample. Then the scan rate was dropped to every 16 min for an additional ~8 h. The data were analyzed using the SEDFIT program to obtain the sedimentation coefficient, c(s). Each distribution was normalized by setting the total area under the curve to 1 (100%) so the area under each peak gave the fraction of that species across the peak. The main peak with the sedimentation coefficient of 1.15 ± 0.04 S corresponded to the pegfilgrastim monomer with a molecular weight of ~40 kDa. The minor peaks (> 1.15 ± 0.04 S) corresponding to the aggregate species were summed up to give the total aggregate %.

### Size exclusion chromatography with multi-angle light scattering (SEC-MALS)

Pegfilgrastim samples were diluted to 1 mg/mL in pegfilgrastim formulation buffer and 50 μL of the diluted sample was injected into a TOSOH TSK GEL G3000 SWXL column (25 nm, 7.8 × 300 mm) connected to Agilent 1260 Infinity LC System. The size variants were separated using 100 mM sodium phosphate buffer containing 10% ethanol, pH 6.4, and detected at 280 nm keeping the column temperature at 30°C with a flow rate of 0.5 mL/min. A specific refractive index increment, dn/dc, of 0.172 mL/g for Pegfilgrastim and 0.134 mL/g for PEG was used for molecular weight estimation.

### Cation exchange chromatography (CEX-HPLC)

Pegfilgrastim samples were diluted to 5 mg/mL and 20 μL of diluted samples were injected into a PolyLC Sulfoethyl A column (1000 Å, 200 × 2.1 mm) connected to Agilent 1260 Infinity LC System. The charge variants were separated by applying a linear gradient from 5 to 40% of mobile phase B (20 mM ammonium acetate, 5% isopropanol, 500 mM ammonium chloride, pH 5.0) with mobile phase A (20 mM ammonium acetate, 5% isopropanol, pH 5.0), and detected at 280 nm keeping the column temperature at 30°C with a flow rate of 0.25 mL/min. The peak distribution was obtained using Chromeleon^TM^ software version 6.8.

### Reverse phase HPLC (RP-HPLC)

Pegfilgrastim samples were diluted to 1 mg/mL and 25 μL of diluted samples were injected into a Chromasol Jade C4 column (300 Å, 4.6 mm × 150 mm) connected to Agilent 1260 Infinity LC System. The hydrophobic variants were separated by applying a linear gradient from 40 to 80% of mobile phase B (90% acetonitrile with 0.1% trifluoroacetic acid) with mobile phase A (0.1% trifluoroacetic acid in water), and detected at 215 nm keeping the column temperature at 60°C with a flow rate of 0.8 mL/min. The peak distribution was obtained using Chromeleon^TM^ software version 6.8.

### Size exclusion UPLC (SE-UPLC)

Pegfilgrastim samples were diluted to 0.25 mg/mL and 2 μL of the diluted samples was injected into acquity SE-UPLC column (450 Å; 4.6 × 150 mm) connected to Waters ACQUITY UPLC H-Class System. The variants were separated using 0.1% formic acid and 0.1% trifluoroacetic acid in 30% acetonitrile and detected at 214 nm keeping the column temperature at 25°C with a flow rate of 0.46 mL/min. The obtained chromatograms were analyzed to obtain the percentage of pegylated variants (dimer pegfilgrastim, dipegylated pegfilgrastim, pegfilgrastim, and filgrastim moieties) using Xcalibur software.

### RP-UPLC with charged aerosol detection (RP-UPLC-CAD)

Pegfilgrastim samples (100 μg) were injected into a Mabpac RP column (1500 Å, 3.0 × 100 mm). The peaks were separated using Thermo Scientific Vanquish UHPLC System by applying a linear gradient from 33 to 70% of mobile phase B (0.1% trifluoroacetic acid in acetonitrile) with mobile phase A (0.1% trifluoroacetic acid in water) for 15 min, keeping the column temperature at 50°C with a flow rate of 0.3 mL/min. The evaporator temperature was set at 50°C, and gas regulation mode was kept as analytical for the CAD detector. Quantitation of PEG was done using the linear plot of PEG standards from 5 to 50 μg/mL.

### Protein quantification

Protein samples were diluted 15 to 20 fold with 5% D-sorbitol solution. Abs_280_ was measured using Agilent Technology carry 60 UV-VIS for three independent preparations. The protein concentration in mg/mL was calculated using the formula Abs_280_ × Dilution factor/0.86.

### Extinction coefficient determination (Edelhoch)

Absorbance of folded and unfolded protein was measured at 280 nm using Agilent Technology carry 60 UV-VIS. For absorbance of folded (native) protein (A_F_), pegfilgrastim was diluted to 0.5 mg/mL in 20 mM potassium phosphate monobasic buffer (pH 6.5); and for absorbance of unfolded protein (A_U_), pegfilgrastim was diluted to 0.5 mg/mL with 6.6 M guanidine hydrochloride in 40 mM potassium phosphate buffer (pH 6.5). The value of Ɛ_U_ for the unfolded protein at 280 nm was calculated using the formula, ${Ɛ}_{U}=(X\times n_{\mathrm{Trp}})+(Y\times n_{\mathrm{Tyr}})+(Z\times n_{s‐s})$ and the molar extinction coefficient of the native protein (Ɛ_F_) was determined using the formula, Ɛ_F =_ Ɛ_U X_ (A_F_/A_U_).

### Micro-flow imaging (MFI)

Contents of the pegfilgrastim drug product were transferred into particle-free tubes for loading to the sample port of a ProteinSimple MFI 5200 instrument. First, 0.2 mL (~10 mg/mL) of the sample was purged through the flow cell and then 0.26 mL was analyzed for sub-visible particles using MVSS software version 2-R4.2.0.42.5211. Following analysis, the morphological characterization of the particle images was performed by MFI View Analysis Suite (MVAS) version 1.4.0. Using optical filters, the silicone oil population was separated from the protein particle population.

### Forced-degradation studies

Three batches of Lupin’s Pegfilgrastim and Neulasta® were subjected to stress conditions for forced-degradation studies. Samples were subjected to oxidative, photolytic, mechanical, pH (low and high), and thermal stress.

For oxidative stress, samples diluted to 1 mg/mL were incubated with 0.01% H_2_O_2_ (v/v) at 25°C for 0, 1, 2, 3, and 5 days. For photolytic stress, undiluted samples were exposed to light at 0, 25, 50, and 100% exposure at 25°C (Osworld Photostability Chamber). For mechanical stress, undiluted samples were agitated at 300 rpm using Eppendorf Thermomixer C at 25°C for 0, 1, 3, 5, and 7 days. For low pH stress, samples diluted to 1 mg/mL were exposed to pH 2.0 using 20 mM glycine hydrochloride at 25°C for 0, 3, 7, 15, and 30 days. For high pH stress, samples diluted to 1 mg/mL were exposed to pH 9.0 using 20 mM Tris-hydrochloride buffer at 25°C for 0, 1, 2, 3, and 7 days. For thermal stress, undiluted samples were exposed to 40°C and 75% humidity for 0, 1, 3, 7, 15, and 30 days (Thermolab Scientific Stability Chamber). The force-degraded samples were analyzed by SE-HPLC, CEX-HPLC, RP-HPLC, RP-UPLC-CAD, and MNFS-60 cell-based potency assay. RP-UPLC-CAD was not performed for the samples subjected to oxidative stress.

### Statistics

A risk-based CQA ranking approach was adopted. Based on the criticality and as per FDA’s guidance on the development of therapeutic protein biosimilars, a data evaluation plan was designed [14]. The risk ranking approach used in CQA assessment encompasses two factors, ‘Impact’ and ‘Uncertainty’. The score for impact was assigned on a scale of 2 to 20 (Table 1). The uncertainty score was determined by the relevance of knowledge used in impact assessment. Based on the relative confidence in information about an attribute’s impact, uncertainty was ranked on a scale of 1 (lowest) to 7 (highest). The ‘Risk’ score was calculated by multiplying Impact and Uncertainty. Product quality attributes were further categorized into different criticality levels based on the risk scores as follows: ≥ 20, very high criticality; 16 to 19, high criticality; and 12 to 15, moderate criticality [15].

**Table 1:** Criticality level of attributes analyzed.

Attribute	Risk score	Criticality
Potency	20	Very high
Receptor Binding	20	
Primary, secondary and higher order structure	20	
Aggregates (high molecular weight)	32	
Protein content	20	
Sub-visible particles	20	
Oxidized species	16	High
Pegylated variants	16	
Free filgrastim/Des-PEG (low molecular weight)	16	
Free m-PEG	16	
N-terminal truncated species	12	Moderate
Polydispersity	12	

PEG, polyethylene glycol

After risk ranking, the quantitative attributes of high and moderate risks such as critical functional assays and protein content were compared using a quality range (QR) approach, 1-sided or 2-sided [16, 17]. The upper and lower limits of QR were derived as mean±2 standard deviation (SD) from the reference product. Product-related impurities were considered for a one-sided comparison and the upper limit was derived as mean+2 SD from the reference product.

In addition to QR evaluation, equivalence testing was performed for some of the very high risk score (≥20) critical functional assays and protein content [16, 17]. A two-sided 95% confidence interval (CI) was obtained for the difference of means with a pre-defined equivalence margin of 2σR (σR was the variability of the reference product) derived from 12 batches of the reference product once normality and equal variances were demonstrated at a p>0.05 significance level. The equivalence analysis was performed using Minitab 17.0.

Principal component analysis (PCA) was performed using JMP version 16.

## Results and discussion

### Structural analyses

We have employed a comprehensive set of orthogonal methods to compare the structural similarity of Neulasta® and Lupin’s Pegfilgrastim at four levels of structural complexity—primary, secondary, tertiary, and higher order structure [14, 18].

Primary structure is one of the foremost attributes for similarity assessment as it provides an unequivocal identity to the molecule. We compared the primary structure of Neulasta® and Lupin’s Pegfilgrastim by Western blotting, peptide mapping, amino acid sequencing, amino acid composition analysis, intact mass analysis, and size and polydispersity analysis.

Western blotting showed that the anti-filgrastim and anti-PEG antibodies detected Neulasta® and Lupin’s Pegfilgrastim and the electrophoretic mobility of both the samples was comparable (S1 Fig).

**Fig 1:**
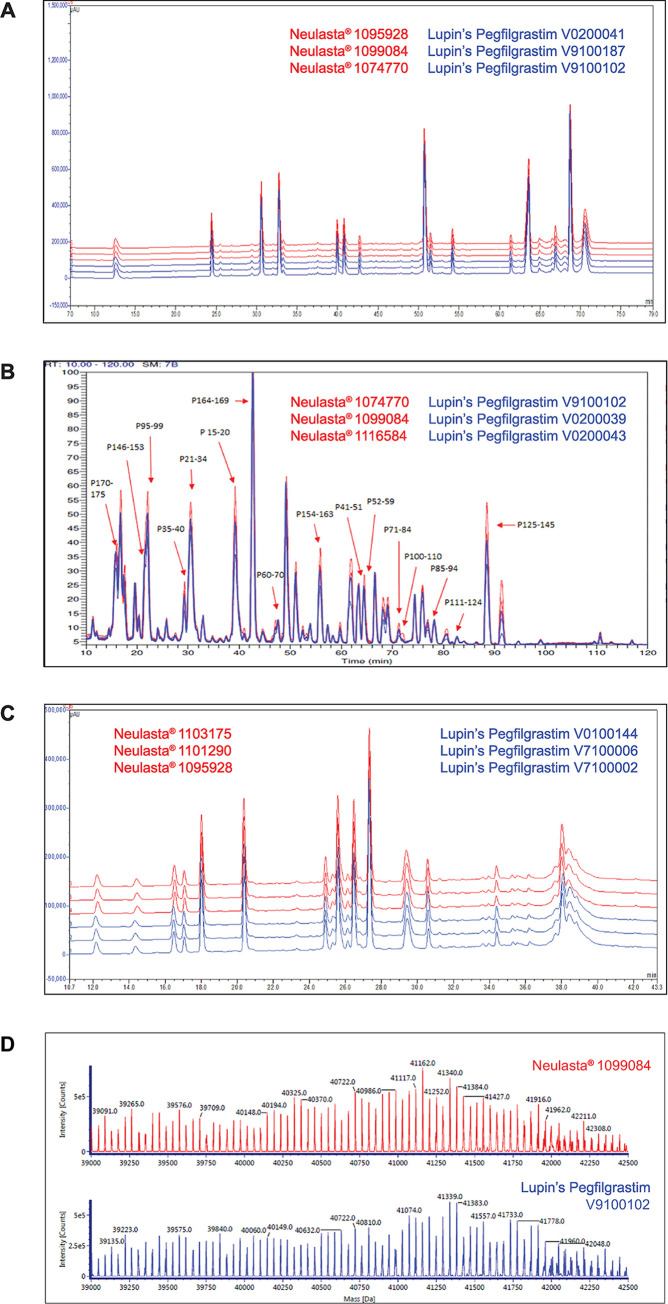
Comparison of chromatographic profiles. (A) Reduced peptide mapping by UV detection, (B) Reduced peptide mapping by MS analysis (with identified peptides annotated) (C) Non-reduced peptide mapping by UV detection (D) Deconvoluted mass spectra for intact mass and polydispersity analysis (Representative images).

Peptide mapping elucidates the primary amino acid structure of protein and provides information regarding protein modifications [19]. Reduced peptide mapping by UV detection and MS analysis and non-reduced peptide mapping by UV detection gave visually superimposable chromatograms for Neulasta® and Lupin’s Pegfilgrastim (Fig 1).

Likewise, reduced peptide mapping by MS analysis gave peptide sequences and fragment masses that were comparable for the two products (S1 Table).

Amino acid composition analysis showed that Neulasta® and Lupin’s Pegfilgrastim had similar amino acid compositions. All the amino acids quantified for Lupin’s Pegfilgrastim were within the generic acceptance range of 85 to 115% of the mean value for Neulasta® (S2 Table).

Pegfilgrastim has covalently linked PEG molecules that are heterogeneous in nature. PEG molecules showed molecular weight distribution in the range of about 18 to 22 kDa with a peak apex at 20 kDa. The attached PEG is heterogeneous with respect to +44 Da PEG polymers. This inherent polydispersity of PEG required charge stripping of PEG molecules before ESI-based MS analysis. Charge stripping of pegfilgrastim by a post-column addition of triethylamine (TEA) simplified the mass spectrum. Thus, all individual pegfilgrastim (+44 Da) mass isoforms were resolved, and the polydispersity, *i*.*e*., distribution of individual pegfilgrastim isoforms, was calculated. The intact molecular weight of the protein was estimated using LC-MS wherein the ionized molecules were separated based on the size of the molecular ion and charge followed by deconvolution (Fig 1D) [20]. The mean intact mass (Neulasta® = 40488.2 Da and Lupin’s Pegfilgrastim = 40533.9 Da) and the mean polydispersity index (Neulasta® = 1.0004 and Lupin’s Pegfilgrastim = 1.0003) were similar for both the products (S3 Table).

We also analyzed the N-terminal methionine pegylation (using LC-MS) after one cycle of Edman degradation [21]. This method gave de-pegylated filgrastim without N-terminal methionine residue (des-PEG-Met filgrastim) and PEG-Met moiety. Intact protein analysis of des-PEG-Met filgrastim and released PEG-Met moiety, and peptide mapping analysis of des-PEG-Met filgrastim were performed. This provided the size and polydispersity of the intact molecule (des-PEG-Met filgrastim) and the released PEG molecule. The mean intact mass (Neulasta® = 22061 Da and Lupin’s Pegfilgrastim = 22090 Da) and the mean polydispersity index of the released PEG-Met moiety (Neulasta® = 1.00063 and Lupin’s Pegfilgrastim = 1.0007) after one cycle of manual Edman degradation were similar for both the products (S4 Table). The masses for des-PEG-Met filgrastim and other respective phenylthiocarbamyl adducts were similar across all tested batches of Neulasta® and Lupin’s Pegfilgrastim (S5 Table). Peptide mapping after one cycle of Edman degradation also showed similar peptides between the products (S6 Table). Furthermore, it also confirmed the pegylation site of filgrastim (N-terminal methionine site).

**Fig 2:**
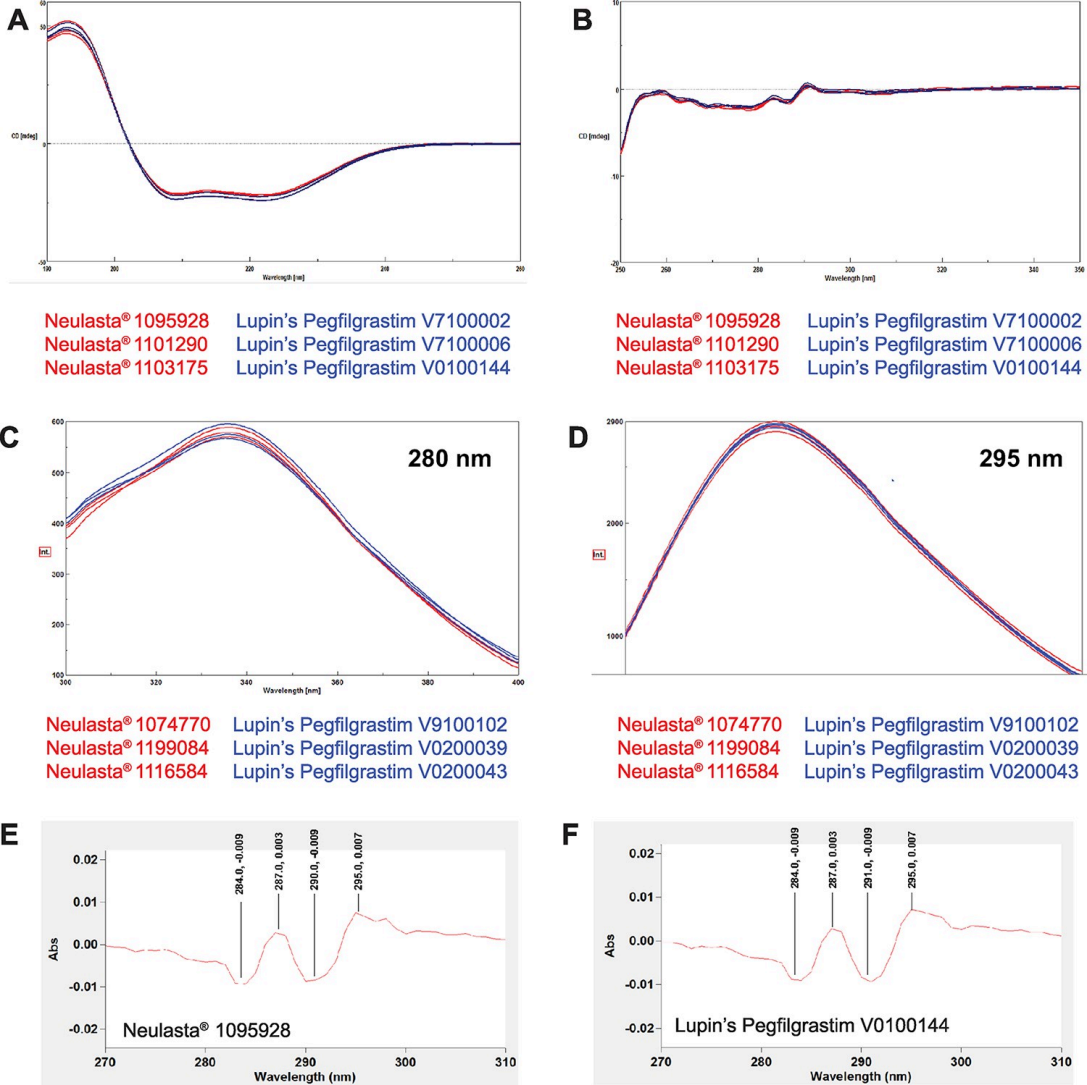
Comparison of spectral profiles. (A) Far UV CD spectra, (B) Near UV-CD spectra, (C) Intrinsic fluorescence spectra at 280 nm and (D) 295 nm. 2D UV-visible spectra of (E) Neulasta® and (F) Lupin’s Pegfilgrastim (Representative images).

After confirming the primary structure, we used multiple structure analysis tools for the comparison of the gross secondary and tertiary structures. The secondary structure of both pegfilgrastim products was compared using Far UV CD and FTIR. Far UV CD measures the α-helix, β-sheet, and random coil structure content within a protein [22]. Neulasta® and Lupin’s Pegfilgrastim gave superimposable spectra in the far UV range (Fig 2A); the percentages of helix, parallel sheet, beta turn, and random coil were also comparable (Table 2).

**Table 2:** Comparison of secondary structure analyzed by Far UV CD.

Sample	% Helix (mean±SD)	% Parallel (mean±SD)	% Beta Turn (mean±SD)	% Random coil (mean±SD)
**Neulasta® (n = 6)**	96.2±0.3	2.1±0.4	0.9±0.4	0.7±0.4
**Lupin’s Pegfilgrastim (n = 12)**	96.4±0.6	2.2±0.2	0.7±0.2	0.5±0.2

n, number of batches; SD, standard deviation

FTIR provides information on local conformational changes within a protein[23]. The similarity in secondary structure was further confirmed by using the correlation between protein structure and IR band frequencies of the amide I region (1700 to 1600 cm^-1^) which is mainly associated with the C = O stretching vibration of a peptide bond and hence is directly related to the protein backbone conformation [24]. The mean IR wave number of the amide I region was similar for both the products (Neulasta® = 1654.0 cm^-1^ and Lupin’s Pegfilgrastim = 1653.6 cm^-1^). The second derivative FTIR spectra were also similar for both the products (S2 Fig).

Further, we compared the tertiary structure of both pegfilgrastim products using near UV CD, intrinsic fluorescence spectroscopy, and second derivative UV-visible spectroscopy. The CD spectra in the near UV range (260 to 320 nm) are a result of the aromatic amino acids tryptophan, tyrosine, and phenylalanine present in the sequence. These spectra help in assessing the protein conformation, and hence, provide insights into the tertiary structure of the protein [22]. Neulasta® and Lupin’s Pegfilgrastim gave superimposable spectra in the near UV range (Fig 2B).

Intrinsic fluorescence spectroscopy provides gross three-dimensional structural information of the protein by measuring the fluorescence of a protein due to intrinsically fluorescent aromatic amino acids [25]. The samples were excited at 280 nm and 295 nm (Emission range 300 to 400 nm for both excitation wavelengths). The intrinsic fluorescence spectra were similar for both the products (Fig 2C and 2D).

Second derivative UV-visible spectroscopy is used to study the conformational effects of solvents on proteins. Derivatization of UV-visible spectra reveals the fine structure of the protein [26]. The derivatized UV-visible spectra were similar for both the products (Fig 2E and 2F).

Next, we compared the higher order structure of both pegfilgrastim products using disulfide bridge analysis, differential scanning calorimetry, and NMR.

Disulfide bond pattern is critical for the three-dimensional structure and function of a protein [27]. We assessed the disulfide bonds and free cysteine residues under non-reducing conditions using LC-MS after proteolytic digestion of pegfilgrastim. The observed masses of respective peptides were similar for Neulasta® and Lupin’s Pegfilgrastim (Table 3). The free cysteine at the C18 position, and the C37-C43 and C65-C75 disulfide bonds were similar for both the products (Table 3).

**Table 3:** Comparison of disulfide bond mapping.

Disulfide bond/ Free Cysteine	Sequence	Theoretical Mass (Da)	Neulasta^®^ 1099084	Lupin’s Pegfilgrastim V9100102
			Observed Mass (Da)	
C37-C43	KLCATY; KLCHPEE	1549.7269	1549.7539	1549.7554
C65-C75	APLSSCPSQALQLAGCLSQLHSGLF	2525.2407	2525.2418	2525.2389
C18	LLKCLE	774.4309	774.4306	774.4309

**Fig 3:**
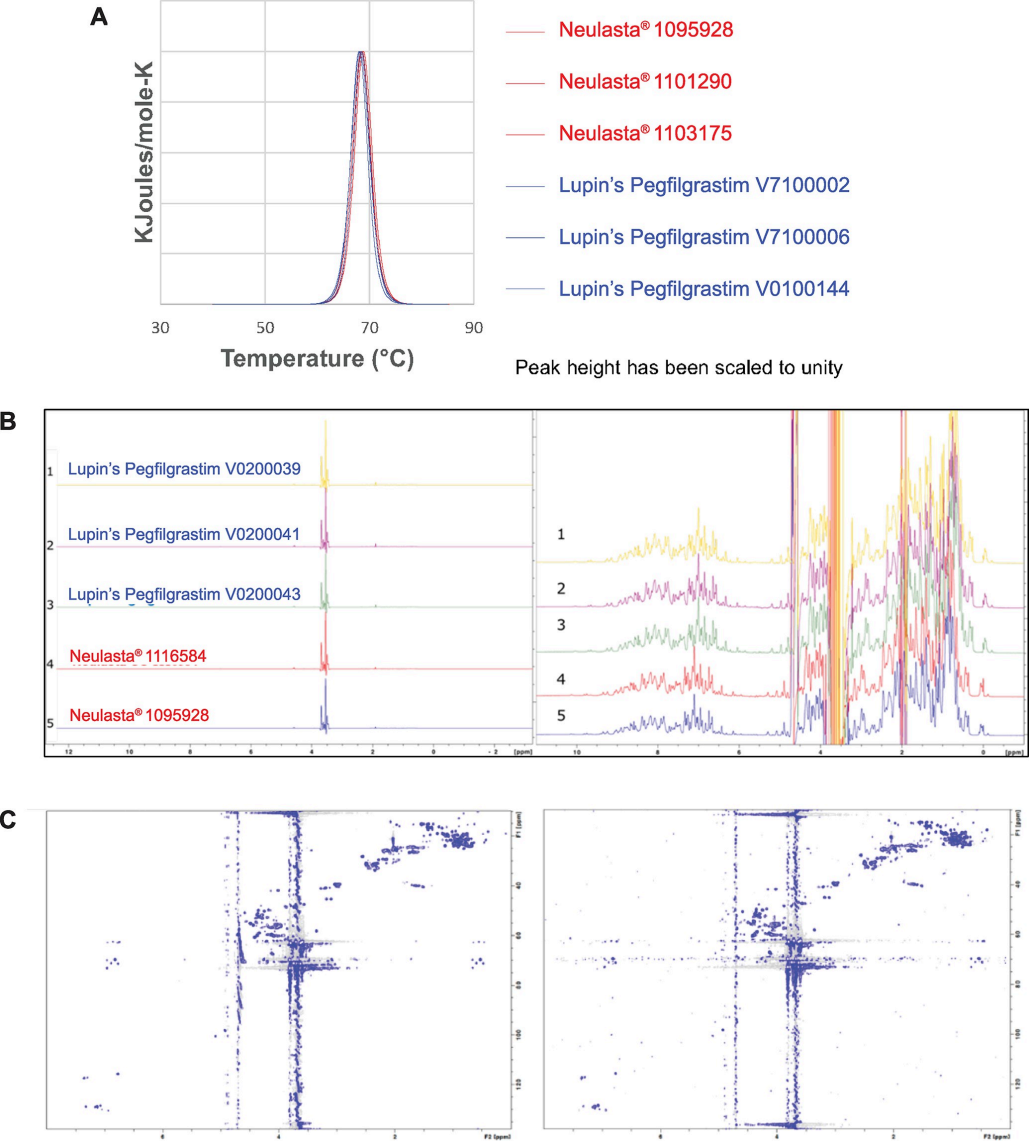
Comparison of higher order structures. (A) Thermal denaturation profiles, (B) 1D ^1^H NMR spectra with a full intensity scale (left panel) and with an expanded intensity scale (right panel), (C) 2D ^1^H-^13^C HSQC NMR spectra at natural abundance of Lupin’s Pegfilgrastim; V0200039 (left panel) and Neulasta® 1095928 (right panel). Cross peaks represent fingerprint C-Hn resonances from the polypeptide and PEG.

Differential scanning calorimetry evaluates the higher order structure of a protein by thermal denaturation [28]. The protein solution is heated and the protein unfolds at a characteristic melting temperature. The mean melting temperature was similar for both the products (Neulasta® = 68.5°C and Lupin’s Pegfilgrastim = 68.4°C). The thermal denaturation profiles of both the products were superimposable (Fig 3A).

The 1D- and 2D-NMR methods were used to obtain a structural fingerprint of the protein that was subsequently used to assess the similarity of the two protein samples [29, 30].

PEG peaked at ~3.65 ppm (Fig 3B). Since all the protein samples had similar heights, the ratio of PEG to protein was similar for all samples. The major component of the sample was a well-folded protein. Also, the dispersion of NMR resonances was similar for all samples. Overall, the spectra for Neulasta® and Lupin’s Pegfilgrastim exhibited highly similar fingerprint patterns across the whole spectral width (Fig 3B).

The 2D ^1^H-^13^C heteronuclear single quantum coherence (HSQC) spectra for Neulasta® and Lupin’s Pegfilgrastim (Fig 3C) confirmed the 1D ^1^H NMR spectra. They exhibited highly similar chemical shift patterns. Peaks for the flexible PEG chain dominated the spectrum at ~3.65 ppm ^1^H ppm and 60 to 70 ^13^C ppm as expected. The noise stripes propagating from these peaks were due to the associated sharp line widths and their high intensities. S3 Fig shows the overlay of fingerprint resonances (aliphatic) from the polypeptide of both products.

Thus, NMR demonstrated identical chemical shift patterns for both proteins indicating that the proteins were indistinguishable from each other.

Overall, these analyses demonstrate that Lupin’s Pegfilgrastim is similar to Neulasta® at the structural level.

### Functional analyses

Structural similarity should translate to functional similarity. We compared the functional attributes of Neulasta® and Lupin’s Pegfilgrastim using an *in vitro* cell proliferation assay and SPR.

**Fig 4:**
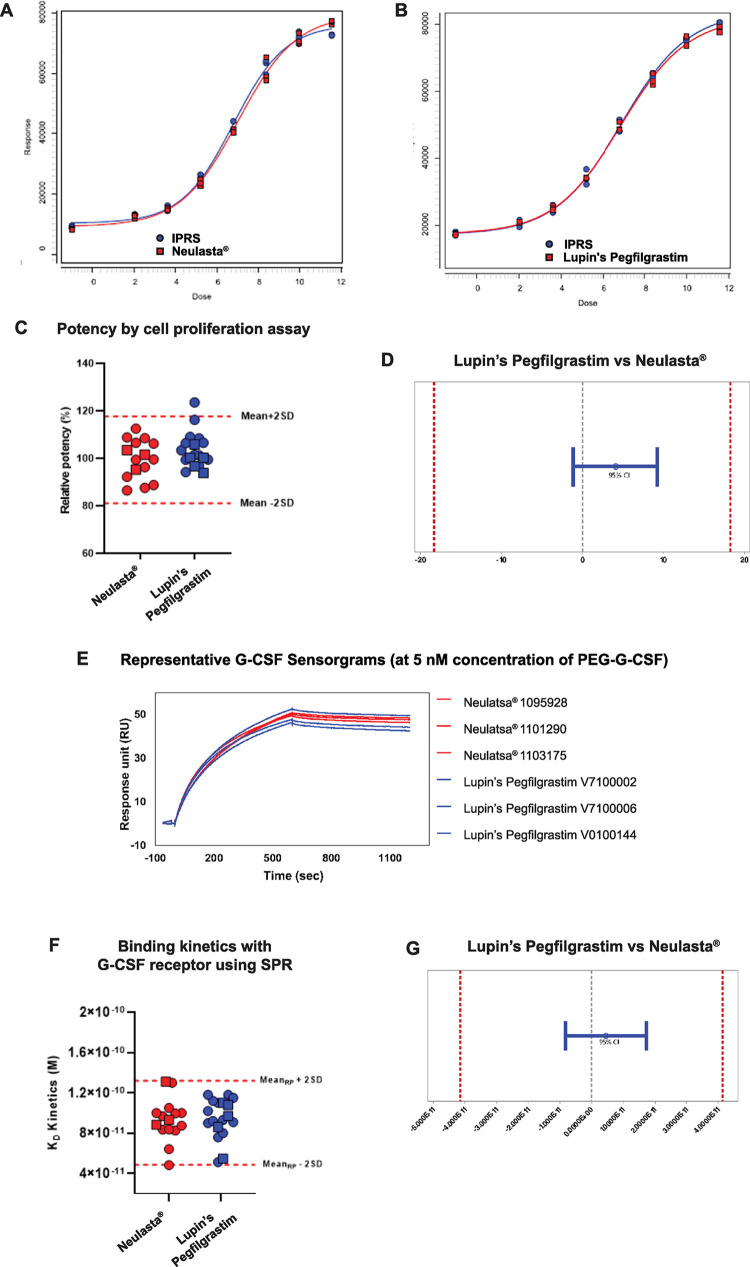
Comparison of functional attributes. Dose response curve of (A) Neulasta® and (B) Lupin’s Pegfilgrastim. (C) Scatter plot of relative potency. (D) Equivalence testing of relative potency. Binding kinetics of pegfilgrastim with G-CSF receptor by SPR (E) G-CSF receptor sensorgrams, (F) K_D Kinetics_ scatter plot, (G) Equivalence testing of receptor binding. The circles and squares represent samples analyzed at two different campaigns.

An *in vitro* cell proliferation assay was employed to determine the biological activity of pegfilgrastim [31]. The dose response curves were similar for Neulasta® and Lupin’s Pegfilgrastim (Fig 4A and 4B). The relative potency scatter plot shows that more than 90% of Lupin’s Pegfilgrastim batches were within mean±2 SD limits of Neulasta® (Fig 4C). One batch had a relative potency marginally above mean±2 SD, but the results of equivalence testing confirmed that the 95% CI of difference of means is within the equivalence margins, determined as 2σ_R_ from Neulasta® (Fig 4D). Also, all Lupin’s Pegfilgrastim and Neulasta® batches comply with the % relative potency specification criteria (Fig 4C). Thus, the *in vitro* biological activity of Lupin’s Pegfilgrastim was similar to that of Neulasta®.

SPR was employed to measure the binding kinetics of pegfilgrastim with the filgrastim receptor [32]. The sensorgrams of Neulasta® and Lupin’s Pegfilgrastim overlapped (Fig 4E). The K_D Kinetics_ scatter plot shows that all batches of Lupin’s Pegfilgrastim were within the mean±2σR limit of Neulasta® (Fig 4F). The results of equivalence testing confirmed that the 95% CI of the difference of means is within the equivalence margins, determined as 2σ_R_ from Neulasta® (Fig 4G). Hence, the binding kinetics of Lupin’s Pegfilgrastim were similar to that of Neulasta®.

Overall, these analyses demonstrate that Lupin’s Pegfilgrastim is similar to Neulasta® at the functional level.

### Purity analyses

The physicochemical characteristics of pegfilgrastim may change due to purification, formulation, packaging, and storage processes [33]. This may yield size variants, charge variants, pegylated variants, or N- and C-terminal variants. Additionally, it may also affect the oxidation and free methoxy-PEG (m-PEG) amount in the final product. We compared these product-related impurities within Neulasta® and Lupin’s Pegfilgrastim using several orthogonal methods [15].

**Fig 5:**
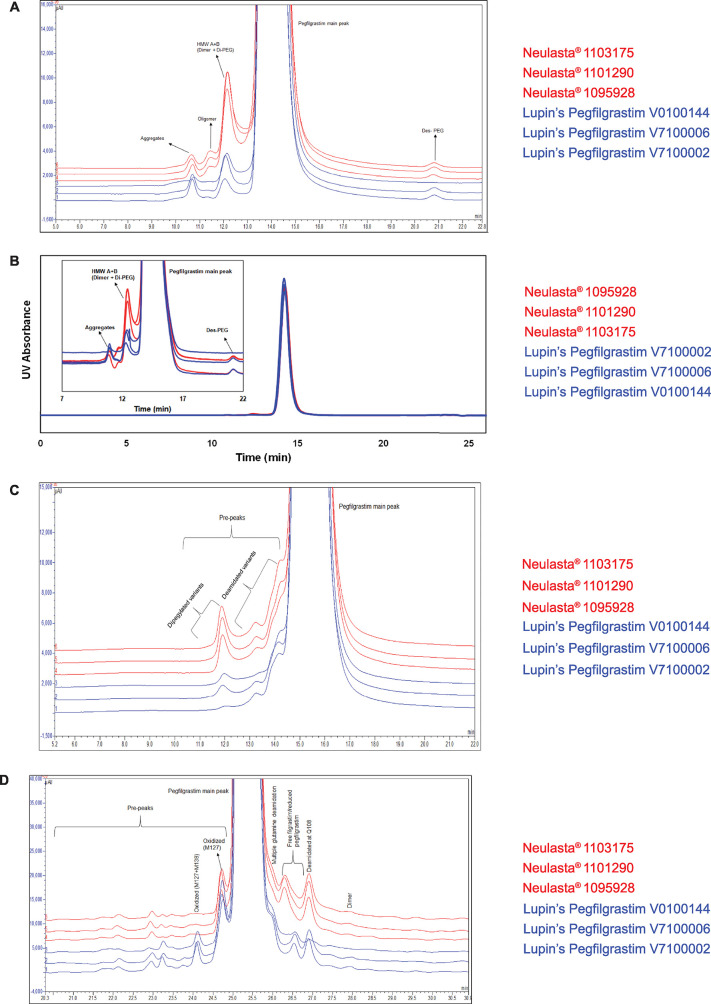
Comparison of product-related impurities. (A) SE-HPLC chromatograms analyzing size variants, (B) SEC-MALS chromatograms analyzing size variants, (C) CEX-HPLC chromatograms analyzing charge variants, (D) RP-HPLC chromatograms analyzing impurities.

**Fig 6:**
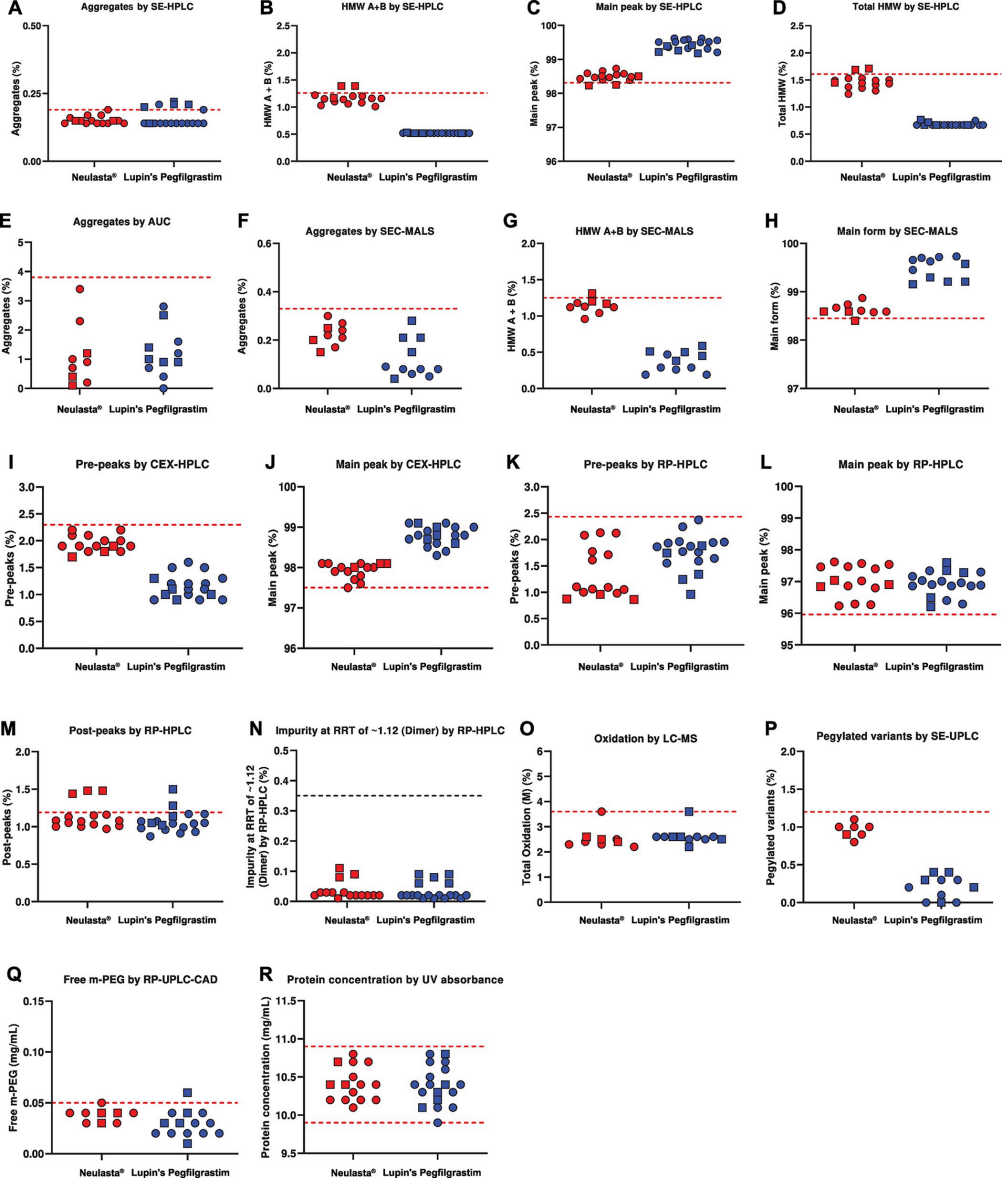
Scatter plots for product-related impurities. (A) Aggregates, (B) HMW A+B, (C) Main peak, and (D) Total HMW measured by SE-HPLC. (E) Aggregates measured by AUC. (F) Aggregates, (G) HMW A+B, and (H) Main form measured by SEC-MALS. (I) Pre-peaks and (J) Main peak measured by CEX-HPLC. (K) Pre-peaks, (L) Main peak, (M) Post-peaks, (N) Dimer measured by RP-HPLC. (O) Total oxidation measured by LC-MS. Observed mass of (P) Pegylated variants measured by SE-UPLC. (Q) Free m-PEG measured by RP-UPLC-CAD. (R) Protein concentration measured by UV absorbance. The circles and squares represent samples analyzed at two different campaigns.

We analyzed pegfilgrastim size variants using SE-HPLC, AUC, and SEC-MALS (Table 4). SE-HPLC separates and quantifies size variants caused by the formation of aggregates, dimers, oligomers, dipegylated forms, free Filgrastim, or Des-PEG. The chromatograms of Neulasta® and Lupin’s Pegfilgrastim were slightly different in terms of the levels; however, the positions of the peaks were comparable (Fig 5A). Lupin’s Pegfilgrastim had similar or slightly lower levels of size variants compared to Neulasta®. Compared to Neulasta®, the percentage of aggregates was marginally higher (Fig 6A); the percentage of the main peak was higher (Fig 6C); and the percentage of high molecular weight species (HMW) A+B (dimer + dipegylated form; Fig 6B and 6D) were lower in Lupin’s Pegfilgrastim (Table 4). Overall, the batches of Lupin’s Pegfilgrastim were comparable to those of Neulasta® with respect to size variants.

**Table 4:** Comparison of product-related variants.

Technique	Characteristic	Neulasta®	Lupin’s Pegfilgrastim
SE-HPLC (Size variants)	n	12	18
	% Aggregates	0.14	0.14
	% HMW (A+B)	1.13	0.37
	% Main peak	98.52	99.43
	% Des-PEG	BQL	BQL
AUC (Size variants)	n	6	11
	% Aggregates	1.4	1.2
SEC-MALS (Size variants)	n	6	11
	% Aggregates	0.23	0.12
	% HMW (A+B)	1.09	0.37
	% Main peak	98.67	99.49
CEX-HPLC (Charge variants)	n	12	18
	% Pre-peak	2	1.2
	% Main peak	97.9	98.8
RP-HPLC	n	12	18
	% Pre-peaks	1.5	1.8
	% Main peak	97	96.9
	% Post-peaks	1.1	1.1
	% Impurity at RRT ~1.07 (Q108)	0.4	0.3
	% Impurity at RRT ~1.12 (Dimer)	BQL	BQL
LC-MS	n	6	11
	% Total oxidation	2.5	2.6
SE-UPLC	n	6	11
	% Pegylated variants	1	0.2
RP-UPLC-CAD	n	6	14
	Free mPEG (mg/mL)	0.04	0.03

n, number of batches tested; SE-HPLC, size exclusion high performance liquid chromatography; AUC, analytical ultracentrifugation; SEC-MALS, size exclusion chromatography with multi-angle light scattering; CEX-HPLC, cation exchange HPLC; RP-HPLC, reverse phase HPLC; LC-MS, liquid chromatography mass spectrometry; SE-UPLC, size exclusion ultra-high performance liquid chromatography; RP-UPLC-CAD, RP-UPLC with charged aerosol detection; HMW (A+B), high molecular weight species dimer + dipegylated form; PEG, polyethylene glycol; RRT, relative retention time; BQL, below quantitation limit

AUC measures the sedimentation coefficient of the protein sample and separates the aggregates or oligomers within the sample. The levels of aggregates were similar for both the products (Fig 6E and Table 4). The marginally higher percentage of aggregates in Lupin’s Pegfilgrastim observed by SE-HPLC was not observed by AUC indicating that these differences were insignificant (compare Fig 6A and Fig 6E).

SEC-MALS estimates the molecular weight of pegfilgrastim and the percentages of size variants present in the sample. There were minor differences in the SEC-MALS chromatograms (Fig 5B). The aggregate levels were similar in both the products (Fig 6F). However, HMW A+B content was slightly lower (Fig 6G) and the main form was higher in Lupin’s Pegfilgrastim batches (Fig 6H and Table 4). SEC-MALS further corroborated the SE-HPLC and AUC data.

We analyzed pegfilgrastim charge variants using CEX-HPLC. CEX-HPLC separates and quantifies differently charged variants such as dipegylated and deamidated variants. The CEX-HPLC chromatograms of Neulasta® and Lupin’s Pegfilgrastim had comparable peak profiles; however, the peak areas were slightly different (Fig 5C). Lupin’s Pegfilgrastim had lower levels of charged variants and concomitantly higher levels of pegfilgrastim (main peak) compared to Neulasta® (Fig 6I, 6J and Table 4).

We analyzed pegfilgrastim product-related variants (impurities) using RP-HPLC. RP-HPLC technique separates product-related impurities (oxidized, reduced, and high molecular weight impurities), product variants generated during the process (dipegylated pegfilgrastim), and free filgrastim based on differences in polarity or hydrophobicity. Neulasta® and Lupin’s Pegfilgrastim showed a similar profile of peaks (Fig 5D). Quantitatively, the peaks were comparable for both the products (Fig 6K to 6M and Table 4). Also, Lupin’s Pegfilgrastim had comparable dimers compared to Neulasta® (Fig 6N and Table 4).

Additionally, we analyzed site-specific pegfilgrastim oxidation and N- and C-terminal variants using LC-MS. The total oxidation and the type of oxidized species were similar for both the products (Fig 6O, Table 4 and S4 Fig).

All the N- and C-terminal variants were below the limit of relative quantitation. Qualitatively, the type of N- and C-terminal variants were same in Neulasta® and Lupin’s Pegfilgrastim (S7 Table).

Next, we analyzed pegylated variants using SE-UPLC. Apart from N-terminal methionine, lysine (K25, K35, or K41) can also be pegylated. The percentage of pegylated variants was lower in Lupin’s Pegfilgrastim than in Neulasta® (Fig 6P and Table 4).

Finally, we analyzed free m-PEG impurities using RP-UPLC-CAD. The free m-PEG content was slightly different between the two products (Fig 6Q and Table 4). However, the difference between the means was statistically non-significant (p = 0.124).

Overall, these analyses demonstrate that Lupin’s Pegfilgrastim is similar to Neulasta® at the purity level. Lupin’s Pegfilgrastim had minor differences with respect to certain impurities when compared to Neulasta®. However, these differences do not impact the function of pegfilgrastim. Lupin’s Pegfilgrastim demonstrated functional similarity to Neulasta® in the G-CSF receptor binding analysis and the *in-vitro* cell proliferation assay. Moreover, these minor product-related attribute differences are not clinically meaningful. In a phase I study in healthy subjects and a phase III study in cancer patients, Lupin’s Pegfilgrastim has demonstrated pharmacokinetic, pharmacodynamic, and therapeutic equivalence to the reference product [9]manuscript under preparation].

### Drug product-related attribute analyses

We further compared Neulasta® and Lupin’s Pegfilgrastim with respect to the protein content, extinction coefficient, and sub-visible particulate matter.

The concentration of protein in the tested lots for Neulasta® (mean concentration = 10.4 mg/mL) and Lupin’s Pegfilgrastim (mean concentration = 10.4 mg/mL), as assessed by UV absorbance at 280 nm, were highly similar (Fig 6R). The extinction coefficient determined by Edelhoch method was also similar for both the products (Lupin’s Pegfilgrastim = 0.838 and Neulasta® = 0.843; S8 Table).

MFI characterizes the sub-visible particles in the pegfilgrastim samples using a high magnification camera that images sub-visible particles (2 to 70 μm) passing through a flow cell. For each sub-visible particle size category, the maximum value obtained for Lupin’s Pegfilgrastim batches was either well within or similar to the maximum value obtained for Neulasta® batches (Table 5). Hence, the sub-visible particle distribution and concentration in Lupin’s Pegfilgrastim was similar to that of Neulasta®.

**Table 5:** Comparison of total sub-visible particles obtained by MFI.

Characteristic			Neulasta®	Lupin’s Pegfilgrastim
n			3	3
Age (months)		Minimum	7	22
		Maximum	35	35
Total particles per mL	2 to 5 μm	Minimum	16104	7804
		Maximum	201387	49785
	5 to 10 μm	Minimum	1597	1200
		Maximum	26804	3593
	10 to 25 μm	Minimum	432	96
		Maximum	921	560
	25 to 50 μm	Minimum	15	15
		Maximum	73	106
	≥50 μm	Minimum	0	0
		Maximum	4	0

n, number of batches tested

### Forced-degradation studies

The presence of impurities in the biologic or biosimilar can affect its stability during transport and storage. Forced-degradation studies or stability studies under stress conditions employ different stress conditions to speed up the degradation pathways and can provide insight into the changes that a therapeutic would undergo under storage conditions [34]. Moreover, forced-degradation studies are important for a direct stability comparison of the biosimilar with the reference product [14].

Even though Lupin’s Pegfilgrastim is analytically similar to Neulasta®, the minor differences in impurities may potentially modify the protein during storage. To evaluate this, we performed comparative forced-degradation studies by subjecting Neulasta® and Lupin’s Pegfilgrastim to various stress conditions like light, heat, acid/base, shear, and chemical modifiers, and subsequently comparing degradation kinetics between the two products. The stress conditions employed may potentially modify the molecular structure of pegfilgrastim. To evaluate this, the force-degraded samples were analyzed using the methods employed to assess analytical similarity. Under all stress conditions tested, the species observed in Lupin’s Pegfilgrastim were also observed in Neulasta®. The rates of degradation were also comparable between the two products, and differences, if any, were marginal (Table 6 and S3 Appendix).

**Table 6:** Summary of forced-degradation studies.

Analytical method	Observations	Remarks
Oxidative stress (0.01% H_2_O_2_) at 25°C		
RP-HPLC	Increase in pre-peak impurities and total related proteins observed. Significant increase in oxidized impurities at RRT of ~0.98 (M127) and ~0.99 (M138). Rate of change in oxidized impurities was linear till day 3 and showed an exponential increase at day 5.	Similar degradation pathway for both the products.
SE-HPLC	Gradual increase in HMW species (aggregates) observed. Rate of aggregate formation was linear.	Similar degradation pathway for both the products.
CEX-HPLC	Increase in post-peak impurities observed. Rate of post-peak impurities formation was linear. Comparable total charge variants at day 5 and similar degradation pathway.	Similar degradation pathway for both the products.
Cell proliferation assay	No impact on potency.	Similar functionality.
Low pH exposure (pH 2.0) at 25°C		
RP-HPLC	Increase in pre-peak and post-peak impurities. Significant increase in oxidized impurity at M127 position (RRT of ~0.98) and deamidated impurity at Q108 position (RRT of ~1.07). Rate of increase in post-peak impurities was linear.	Similar degradation pathway for both the products.
CEX-HPLC	Increase in pre-peak impurities and total charged variants. Degradation via deamidation. Rate of increase in pre-peak and post-peak impurities was linear. Pre-peak impurities in Lupin’s Pegfilgrastim were lower across all time points.	Similar degradation pathway for both the products.
SE-HPLC	Degradation via aggregation. Rate of aggregate formation was linear. Increase in level of Des-PEG. No change in dimer levels. Total size variants in Lupin’s Pegfilgrastim were lower at each time point.	Similar degradation pathway for both the products.
RP-UPLC-CAD	Free PEG content increased at the last time point as compared to untreated sample.	Similar free PEG content in both the products.
Cell proliferation assay	No impact on potency.	Similar functionality.
High pH exposure (pH 9.0) at 25°C		
RP-HPLC	Rate of increase in post-peak impurities and total related proteins was lower in Lupin’s Pegfilgrastim. Degradation via aggregation and dimerization.	Lupin’s Pegfilgrastim showed slower degradation; similar degradation pathway.
CEX-HPLC	Increase in post-peak impurities and total charged variants observed. Rate of increase in post-peak impurities was lower In Lupin’s Pegfilgrastim.	Lupin’s Pegfilgrastim showed slower degradation; similar degradation pathway.
SE-HPLC	Degradation via aggregation and dimerization. Rate of aggregate formation was marginally lower in Lupin’s Pegfilgrastim.	Lupin’s Pegfilgrastim showed slower degradation; similar degradation pathway.
	No significant change in levels of Des-PEG. Total size variants were lower in Lupin’s Pegfilgrastim at each time point.	
RP-UPLC-CAD	Increased free PEG content at last time point when compared with untreated sample.	Similar free PEG content in both the products.
Cell proliferation assay	No impact on potency.	Similar functionality.
Photolytic degradation (white light exposure) at 25°C		
RP-HPLC	Rate of increase in pre-peak and total related proteins was lower in Lupin’s Pegfilgrastim. Degradation via oxidation.	Lupin’s Pegfilgrastim showed slower degradation; similar degradation pathway.
SE-HPLC	Rate of aggregate formation was marginally lower in Lupin’s Pegfilgrastim. Degradation via aggregation.	Lupin’s Pegfilgrastim showed slower degradation; similar degradation pathway.
CEX-HPLC	Increase in pre-peak, post-peak impurities and total charged variants was lower in Lupin’s Pegfilgrastim.	Lupin’s Pegfilgrastim showed slower degradation; similar degradation pathway.
RP-UPLC-CAD	Free PEG content was lower in Lupin’s Pegfilgrastim at all tested time points.	Lupin’s Pegfilgrastim had lower free PEG content.
Cell proliferation assay	No impact on potency.	Similar functionality.
Photolytic degradation (UV light exposure) at 25°C		
RP-HPLC	Significant increase in pre-peak impurities and total related proteins for both the products. Degradation via oxidation.	Similar degradation pathway for both the products.
SE-HPLC	Degradation via aggregation and dimerization. Significant change in levels of aggregates and dimer, but not Des-PEG.	Similar levels of size variants for both the products; similar degradation pathway.
CEX-HPLC	Significant increase in pre-peak, post-peak impurities and total charged variants for both the products. But pre-peak and post-peak impurities were lower in Lupin’s Pegfilgrastim.	Lupin’s Pegfilgrastim showed slower degradation; similar degradation pathway.
RP-UPLC-CAD	No significant change in free PEG content.	Similar free PEG content in both the products.
Cell proliferation assay	Decrease in potency observed.	Reduced functionality observed for both the products.
High temperature exposure/thermal stress at 40°C and 75% humidity		
RP-HPLC	Gradual and linear increase in pre-peak and post-peak impurities observed. Degradation via oxidation and deamidation.	Similar degradation pathway for both the products.
SE-HPLC	Degradation via aggregation. Significant change in levels of aggregates, but not dimers and Des-PEG. Lupin’s Pegfilgrastim showed lower levels of size variants. Rate of increase in size variants was linear.	Lupin’s Pegfilgrastim showed slower degradation; similar degradation pathway.
CEX-HPLC	Significant increase in post-peak impurities. Pre-peak and Post-peak impurities were lower in Lupin’s Pegfilgrastim.	Lupin’s Pegfilgrastim had lower impurities; comparable rate of degradation; similar degradation pathway.
RP-UPLC-CAD	Significant change in free PEG content at last time point observed.	Similar free PEG content in both the products.
Cell proliferation assay	No impact on potency.	Similar functionality.
Mechanical/Shear stress at 25°C		
RP-HPLC	No significant change in pre-peak and post-peak impurity levels.	Similar degradation pathway for both the products.
SE-HPLC	No significant change in levels of aggregates, dimer and Des-PEG.	Similar degradation pathway for both the products.
CEX-HPLC	No significant change in pre-peak and post-peak impurities.	Similar degradation pathway for both the products.
RP-UPLC-CAD	No significant change in free PEG content.	Comparable free PEG content.
Cell proliferation assay	No impact on potency.	Similar functionality.

RP-HPLC, reverse phase high performance liquid chromatography; SE-HPLC, size exclusion HPLC; CEX-HPLC, cation exchange HPLC; RP-UPLC-CAD, RP-UPLC with charged aerosol detection; RRT, relative retention time; HMW, high molecular weight species; PEG, polyethylene glycol

### Similarity assessment

A risk based CQA ranking approach determines quality attributes that characterize the reference product in terms of structural and functional properties according to their potential clinical impact. It considers the known and potential effect of each attribute on bioactivity, pharmacokinetics, pharmacodynamics, immunogenicity, and safety [35].

We compared the structural, functional, purity, and drug product-related attributes of Neulasta® and Lupin’s Pegfilgrastim for similarity assessment. The attributes were compared visually, or by employing a 1-sided or 2-sided quality range approach for analysis. For very high-risk score attributes, equivalence testing was also performed. Table 7 summarizes the results of all quality attributes to support analytical similarity.

**Table 7:** Results of similarity assessment including details of attributes, analytical methods and the number of batches tested.

Attribute/Analytical method		Evaluation approach	n	Neulasta® range^a^	Test range^b^	Similarity established^c^
**Protein structure**						
Reduced Peptide mapping with UV detection		Chromatographic overlays	6:12	Identical peak profile		Yes
Reduced Peptide mapping with MS analysis		Chromatographic overlays	3:6	Visually superimposable peak pattern		Yes
Non-reduced Peptide mapping with UV detection		Chromatographic overlays	3:6	Visually superimposable peak pattern		Yes
Amino acid sequence by peptide mapping with MS/MS analysis		Comparison of tabulated data/Sequence	1:2	Identical primary amino acid sequence		Yes
Intact mass analysis and polydispersity by LC-MS		Comparison of tabulated data	7:9	40458.3 to 40524.1	40456.4 to 40634.1	Yes
				Similar polydispersity index value		
Western Blot		Comparison of band detected	3:6	Similar position of respective bands		Yes
Amino acid composition		Proportion of different amino acids	6:9	Proportion of different amino acids of Pegfilgrastim is similar		Yes
Edman sequencing of PEG site of attachment and N-terminal pegylation by LC/MS)		Sequence comparison	3:4	Similar observed mass and peptide sequence		Yes
Size and polydispersity of intact molecule and released PEG	Polydispersity index value	Qualitative comparison of mass range and polydispersity index	3:3	Similar polydispersity index value		Yes
	des-PEG-Met Filgrastim			Similar mass		Yes
	Released PEG-Met moiety (Min-max, observed mass Da)			21877.5 to 22252.0	21921.0 to 22295.5	Marginally higher but no impact
Far UV CD		Comparison of α helix (%)	6:12	91.3 to 101.0	95.4 to 97.1	Yes
FTIR		Comparison of wavenumber (cm^-1^)	6:12	1638.3 to 1671.3	1651.0 to 1655.1	Yes
Near UV CD		Spectral comparison	6:12	Visually superimposable spectral profile		Yes
Intrinsic Fluorescence spectroscopy	Excitation at 280nm	Comparison of 330/350 ratio	6:12	1.06 to 1.17	1.11 to 1.12	Yes
	Excitation at 295nm			0.95 to 1.05	0.99 to 1.00	
Second Derivative UV spectroscopy		Comparison of a/b ratio	3:6	0.70 to 0.86	0.72 to 0.80	Yes
Disulfide bridge analysis		Comparison of tabulated data/Sequence	1:1	Identical disulfide bonding pattern		Yes
Differential scanning calorimetry		Comparison of melting temperature (°C)	6:12	61.69 to 75.40	68.01 to 68.82	Yes
3D conformation by NMR		Spectral comparison	3:4	Highly similar spectral profile		Yes
**Functional attributes**						
Relative potency (%) using a cell proliferation assay		Equivalence testing	12:18	-18.3 to 18.3	-1.12 to 9.32	Yes; 94% of Lupin’s Pegfilgrastim batches were within the quality range of Neulasta® and 95% CI of the difference of means was within the equivalence margins.
		Quality range		81.1 to 117.7	93.9 to 123.6	
Binding kinetics with filgrastim receptor using SPR (M)		Equivalence testing	12:18	-4.14E-11 to 4.14E-11	-8.16E-12 to 1.75E-11	Yes
		Quality range		4.86E-11 to 1.32E-10	5.12E-11 to 1.18E-10	
**Product-related impurities**						
Size variants by SE-HPLC (HMW and LMW impurities)	Aggregate (%)	Quality range, 1-sided comparison	12:18	≤0.20	0.07 to 0.22	Marginally higher value in Lupin’s Pegfilgrastim but no impact considered as orthogonally (AUC and SEC MALS) demonstrated to be similar.
	HMW A+B (%)			≤1.26	0.21 to 0.53	Yes
	Main peak (%)			≥98.31	99.18 to 99.62	Yes
	Des-PEG (%)			BQL		Yes
Size variants by AUC (%)		Quality range, 1-sided comparison	6:11	≤3.8	0.0 to 2.8	Yes
Size variants by SEC-MALS	Aggregate (%)	Quality range, 1-sided comparison	6:11	≤0.33	0.04 to 0.28	Yes
	HMW A+B (%)			≤1.25	0.19 to 0.59	
	Main peak (%)			≥98.45	99.16 to 99.73	
Charge variants by CEX-HPLC	Pre-peaks (%)	Quality range, 1-sided comparison	12:18	≤2.3	0.9 to 1.6	Yes
	Main peak (%)			≥97.5	98.3 to 99.1	Yes
Related protein variants by RP-HPLC	Pre-peaks (%)	Quality range, 1-sided comparison	12:18	≤2.43	0.96 to 2.37	Yes
	Main peak (%)			≥95.96	96.20 to 97.60	Yes
	Post-peaks (%)			≤1.19	0.87 to 1.50	Post-peak values of two batches marginally outside the range; however, similar values observed in Neulasta® batches analyzed simultaneously but not considered for range determination. Therefore, considered similar.
	Impurity at RRT of ~1.07 (Deamidated at Q108 position) (%)			≤0.52	0.21 to 0.42	Yes
	Impurity at RRT of ~1.12 (Dimer) (%)			BQL		Yes
Oxidation of methionine by LC-MS/MS (%)		Quality range, 1-sided comparison	6:11	≤3.6	2.2 to 3.6	Yes
Pegylated variants by SE-UPLC (%)		Quality range, 1-sided comparison	6:11	≤1.2	0 to 0.4	Yes
N- and C-terminal variants by LC-MS/MS (%)		Quality range, 1-sided comparison	7:11	Below limit of relative quantitation, observed masses comparable		Yes
**Process-related impurities**						
Free m-PEG by RP-UPLC-CAD (mg/mL)		Quality range, 1-sided comparison	6:14	≤0.05	0.01 to 0.06	Yes; 94%of Lupin’s Pegfilgrastim within the Neulasta® range.
**Drug product-related attributes**						
Protein content by UV absorbance (mg/mL)		Equivalence testing	12:18	-0.5 to 0.5	-0.16 to 0.16	Yes
		Quality range		9.9 to 10.9	9.9 to 10.8	Yes
Extinction coefficient determination (Edelhoch)		Comparison of Extinction coefficient values	3:9	0.76 to 0.93	0.82 to 0.86	Yes
Sub-visible particulate matter by MFI (Total particles /mL)		Comparison of particle concentration	3:3	Similar particle concentration		Yes

n, number of batches tested (Neulasta®: Lupin’s Pegfilgrastim); MS, mass spectrometry; LC-MS, liquid chromatography MS; PEG, polyethylene glycol; CD, circular dichroism; FTIR, Fourier transform infrared spectroscopy; NMR, nuclear magnetic resonance; SPR, surface plasmon resonance; SE-HPLC, size exclusion high performance liquid chromatography; HMW, high molecular weight species; LMW, low molecular weight species; HMW (A+B), high molecular weight species dimer + dipegylated form; AUC, analytical ultracentrifugation; SEC-MALS, size exclusion chromatography with multi-angle light scattering; CEX-HPLC, cation exchange HPLC; RP-HPLC, reverse phase HPLC; RRT, relative retention time; SE-UPLC, size exclusion ultra-high performance liquid chromatography; RP-UPLC-CAD, RP-UPLC with charged aerosol detection; MFI, micro-flow imaging

^a^Neulasta® Equivalence margin/ Quality range (Mean± 2 standard deviation)

^b^Lupin’s Pegfilgrastim 95% confidence interval of difference of means/ Min-Max range

^c^Neulasta® vs Lupin’s Pegfilgrastim: Within equivalence margin/Within quality range/ Visual comparison

S9 Table gives an additional descriptive statistics summary and S10 Table gives a summary of the analytical assessment of the difference of means for ‘high to very high’ risk scoring attributes. S4 Appendix gives the PCA using the quantifiable descriptors of the CQA methods. In the purity analyses, except for aggregates, Lupin’s Pegfilgrastim showed lower amounts of variants than Neulasta®. For the rest of the CQAs, both the products were highly similar.

Analytical similarity assessment conclusions and statistical inferences depend on the product lots selected for comparisons [36]. We have selected 12 random lots of Neulasta® and 18 available lots of Lupin’s Pegfilgrastim with variable manufacturing and expiry dates to ensure accurate and reliable quantitation of product variability.

Multiple pegfilgrastim biosimilars have received approval in several countries. Previous studies comparing pegfilgrastim biosimilar with the reference product have used the orthogonal methods we have used in our study [37, 38]. However, this study employs a greater number of methods compared to previously published studies. Brokx *et al*. have also employed *in vivo* studies in mice to demonstrate comparable bioactivity [37]. We have limited our functional analyses to cell proliferation assay and binding kinetics of pegfilgrastim with the filgrastim receptor which are established techniques to assess functional attributes for demonstrating analytical similarity [15].

As of December 2022, FDA has approved 40 biosimilars—several products like adalimumab, trastuzumab, and pegfilgrastim for multiple sponsors, while some like infliximab, ranibizumab, and etanercept for fewer sponsors [39]. Most published biosimilar analytical studies have reported physicochemical properties, impurities, biological activity, and primary structure, only 58% of them reported higher order structures and 28% reported all quality attributes relevant to the active therapeutic protein [40]. Studies reporting forced degradation studies are limited to thermal stress or forced oxidation; no study has included comprehensive forced degradation analysis which is a useful tool for assessing biosimilarity [41–43]. Our study includes all quality attributes relevant to pegfilgrastim, multiple conditions for forced degradation study, and statistical analyses and can provide guidance for establishing analytical similarity studies for this product.

## Conclusion

This study demonstrates the analytical similarity of Lupin’s Pegfilgrastim and Neulasta®. The conclusions drawn from this study are validated by the robust study design, state-of-the-art techniques, and statistical analyses.

In all physicochemical and functional methods, comparable profiles were observed for Neulasta® and Lupin’s Pegfilgrastim. The protein sequence of Lupin’s Pegfilgrastim was identical to that of Neulasta®. The site of pegylation and the size of PEG moiety in Lupin’s Pegfilgrastim and Neulasta® were comparable. Concomitantly, the secondary, tertiary, and higher order structure by multiple orthogonal techniques were also similar for the two products. The size variants, charge variants, and hydrophobic variants quantified by orthogonal techniques were comparable between Lupin’s Pegfilgrastim and Neulasta®. The two products had equivalent binding kinetics with the G-CSF receptor as determined by SPR; they also had equivalent *in-vitro* efficacy as observed by the cell proliferation assay performed using M-NFS-60 cells.

In the comparative forced-degradation study under various stress conditions, no new impurities were observed in Lupin’s Pegfilgrastim; the rate of degradation of Lupin’s Pegfilgrastim was comparable to that of Neulasta®, and the impact on the functional assay was also comparable.

The orthogonal methods, forced-degradation study, and statistical analyses described here not only demonstrate the bioanalytical similarity of Neulasta® and Lupin’s Pegfilgrastim but also provide a framework for designing such studies for pegfilgrastim biosimilars.

## Supporting information

S1 Appendix Supplementary materials. (DOCX)

S2 Appendix Controls used for the methods. (DOCX)

S3 Appendix Forced-degradation study results. (DOCX)

S4 Appendix Principal component analysis (PCA). (DOCX)

S1 Fig Western blot analysis. (DOCX)

S2 Fig FTIR spectra. (DOCX)

S3 Fig Fingerprint methyl/methylene regions from 2D ^1^H-^13^C HSQC NMR spectra at natural abundance of pegfilgrastim samples. (DOCX)

S4 Fig Scatter plot of oxidation by LC-MS. (DOCX)

S1 Table Comparison of theoretical mass and observed mass for corresponding peptide sequence of representative batches of Neulasta^®^ and Lupin’s Pegfilgrastim. (DOCX)

S2 Table Comparison of the amino acid composition of Neulasta® and Lupin’s Pegfilgrastim. (DOCX)

S3 Table Comparison of the intact mass and polydispersity index of Neulasta® and Lupin’s Pegfilgrastim. (DOCX)

S4 Table Comparison of the intact mass and polydispersity index of released PEG-Met moiety from Neulasta® and Lupin’s Pegfilgrastim. (DOCX)

S5 Table Comparison of theoretical masses for des-PEG-Met filgrastim and other phenylthiocarbamyl adducts. (DOCX)

S6 Table Comparison of theoretical mass and observed mass for corresponding peptide sequence after one cycle of Edman degradation of representative batches of Neulasta^®^ and Lupin’s Pegfilgrastim. (DOCX)

S7 Table Comparison of observed mass of N- and C-terminal variant by LC-MS. (DOCX)

S8 Table Molar extinction coefficient determination (Edelhoch). (DOCX)

S9 Table Descriptive statistics summary. (DOCX)

S10 Table Brief summary of analytical assessment for ‘high to very high’ risk scoring attributes. (DOCX)
