# Supplementary material for: Analytical sameness methodology for the evaluation of structural, physicochemical, and biological characteristics of Armlupeg: A pegfilgrastim biosimilar case study
Source: PLoS One. 2023 Aug 9;18(8):e0289745. doi: 10.1371/journal.pone.0289745 (PMC10411777; doi:10.1371/journal.pone.0289745)
Supplement: S1 Appendix — (DOCX) [file pone.0289745.s001.docx]

**S1 Appendix**

**Supporting Materials**

Following chemicals, reagents, and materials were used for the study:

Amino acid hydrolyzed standard (Waters)

AccQ-Tag ultra-derivatization kit (Waters)

Eluent A (Waters)

Eluent B (Waters)

Hydrochloric acid (HiMedia)

Sodium hydroxide (Sigma)

Bovine serum albumin (Thermo Scientific)

Hellmanex III (Hellma)

Tris (hydroxymethyl) aminomethane (Trizma; Sigma)

Trifluoroacetic acid (Sigma)

Acetonitrile (Merck)

Methylamine hydrochloride (Sigma)

Dithiothreitol (Sigma)

Urea (HiMedia)

Hydrochloric acid (HiMedia)

Milli Q /Purified Water (Millipore)

Endoproteinase Glu-C (sequencing grade; Roche)

Monobasic potassium phosphate (Calbiochem)

Dibasic potassium phosphate (Sigma Aldrich)

Guanidine hydrochloride (Sigma Aldrich)

Trifluoroacetic acid (Sigma Aldrich)

Polysorbate 20 (J.T. Baker)

Sorbitol (Merck; Parteck® SI 400)

Sodium acetate trihydrate (Merck)

Acetic acid (Merck or Rankem)

Glutamyl Endopeptidase (Glu-C; Roche or Promega)

0.2 µm syringe filter

Acrylamide (Sigma)

N, N'-Methylenebisacrylamide (Sigma)

Tris (hydroxy methyl) aminomethane (Sigma)

Glycine (Sigma)

Sodium dodecyl sulphate (Calbiochem)

Ammonium persulphate (Sigma)

N, N, N', N'-Tetramethylethylenediamine (Sigma)

Bromophenol blue (Sigma)

1-Butanol (HiMedia)

Glycerol (Sigma)

Formaldehyde (Merck)

Methanol (Finar Reagents)

Silver nitrate (Qualigens)

Sodium thiosulphate (Merck)

Sodium carbonate (Merck)

Barium chloride (Merck)

1M Iodine solution (Merck)

Spectra multicolor broad range protein ladder (Thermo Scientific)

2-Mercaptoethanol (Sigma)

Tris buffered saline (Serva)

BCIP®/NBT Liquid substrate system (Sigma)

Human G-CSF mAb (Clone 3316)

Mouse IgG1 (R&D Systems)

Immun-Blot goat anti-mouse IgG (H + L)-AP assay kit (Bio-Rad)

Anti-Polyethylene glycol antibody [RM105]

Rabbit monoclonal IgG (Abcam)

Donkey anti-rabbit IgG H&L (Alkaline Phosphatase; Abcam)

Nitrocellulose membrane (Bio-Rad)

Hydrogen peroxide (Merck)

Methionine (Sigma)

Sodium phosphate monobasic (Sigma Aldrich)

Sodium phosphate dibasic (Sigma Aldrich)

Ethanol (Merck)

Sodium acetate trihydrate (Merck)

2-Propanol (Merck)

Ammonium chloride (Sigma)

Ammonium acetate (Sigma Aldrich)

Acetonitrile, ULC/MS grade (Biosolve)

Trifluoro acetic acid, ULC/MS grade (Biosolve)

Polyethylene glycol 20 kDa (NOF)

Albumin standard (Thermo Scientific)

Camphorsulfonic acid, ammonium salt (Sigma)

Human G-CSFR/ CD114/ CSF3R Protein (Sino Biological)

10X HBS-EP+ (Cytiva)

Immobilization kit (1-Ethyl-3-(3-dimethylaminopropyl) carbodiimide (EDC)/N-Hydroxy succinimide (NHS)/Ethanolamine (Cytiva)

10 mM Acetate pH 5.5 (Cytiva)

50 mM Sodium hydroxide (Cytiva)

BIAnormalizing solution (Cytiva)

10 mM Glycine, pH 3.0 (Cytiva)

Pyrogen-free, sterile water for injections (WFI) IP (Nirlife®)

Formic acid (Honeywell or Fluka)

Water (J.T. Baker)

Iodoacetamide (Sigma)

Tert-Butyl hydroperoxide solution (Sigma)

Nap 5 column (GE Healthcare)

Chymotrypsin (Promega)

MOPS sodium salt (Sigma)

RPMI-1640 medium with L-glutamine (Sigma)

Water for Injection (Aculife Healthcare)

Heat Inactivated Fetal Bovine Serum (Gibco)

Fetal Bovine Serum (Gibco)

HEPES (Sigma)

Sodium bicarbonate (Sigma)

Penicillin Streptomycin solution (Gibco)

Phosphate buffered saline (Gibco)

0.5% Trypan blue (Sigma)

CellTiter-Glo Luminescent Cell Viability kit (Promega)

Dimethyl sulfoxide (Sigma)

L-Glutamine (Sigma)

Sodium pyruvate (Sigma)

Sodium hypochlorite solution (Fisher Scientific)

Isopropyl alcohol (Finar Chemicals)

0.22 µm PES bottle top filter (Corning/VWR)

96 well tissue culture plate (Corning/CytoOne)

96 well assay plate, black plate clear bottom (Corning)

96 well dilution plate (Thermo Scientific or Eppendorf)

Sodium acetate trihydrate (Merck)
