# Supplementary material for: Analytical sameness methodology for the evaluation of structural, physicochemical, and biological characteristics of Armlupeg: A pegfilgrastim biosimilar case study
Source: PLoS One. 2023 Aug 9;18(8):e0289745. doi: 10.1371/journal.pone.0289745 (PMC10411777; doi:10.1371/journal.pone.0289745)
Supplement: S2 Appendix — (DOCX) [file pone.0289745.s002.docx]

**S2 Appendix. Controls for the methods.**

| **Method** | **Controls** |
| --- | --- |
| Western blot (anti-filgrastim) | Positive control: Pegfilgrastim reference standard; Negative control: Unrelated protein. |
| Western blot (anti PEG moiety) | Positive control: Pegfilgrastim reference standard; Negative control: Filgrastim. |
| Reduced peptide mapping by RP-HPLC | No interference was observed with protein blank, enzyme blank, digestion buffer, formulation buffer and mobile phase buffer in the region of signature peptides. |
| Non-reduced peptide mapping by RP-HPLC | No interference was observed with blank, protein blank, enzyme blank, digestion buffer, formulation buffer and mobile phase buffer in the region of signature peptides. |
| Amino acid composition by RP-UPLC | No interference was observed with system blank, gradient blank and reaction blank at the retention time of amino acid peaks.  Positive control: Amino acid standards. |
| Second derivative UV spectroscopy | Negative control: Formulation buffer. |
| SE-HPLC | No response at the retention time of pegfilgrastim main peak and impurity peaks was observed in matrix injections. |
|  | Positive control: Pegfilgrastim reference standard; Negative control: Filgrastim and unrelated protein. |
| CEX-HPLC | No response at the retention time of pegfilgrastim main peak and its impurities was observed in matrix injections. |
|  | Positive control: Pegfilgrastim reference standard; Negative control: Filgrastim and unrelated protein. |
| RP-HPLC | No response at the retention time of pegfilgrastim main peak and impurity peaks was observed in matrix injections (formulation buffer). |
|  | Positive control: Pegfilgrastim reference standard; Negative control: Filgrastim and unrelated protein (ranibizumab). |
| RP-UPLC-CAD | There was no response observed from matrix (formulation buffer) in integration window of PEG 20 kDa peak. |
|  | PEG-20 kDa containing sample (pegfilgrastim reference standard) showing response of PEG-20 kDa at retention time ~15.3 min. |
|  | The retention time difference between PEG-20 kDa standard (30 µg/mL) and PEG-20 kDa present in test samples like pegfilgrastim IRS, DS and Neulasta® was less than 0.1 min. |
| Protein quantification | Positive control: Pegfilgrastim reference standard; Negative control: Formulation buffer or placebo samples. |
| Extinction coefficient determination (Edelhoch) | Respective diluents are used for blank corrections. |
| Mass spectrometry | Mass instruments were calibrated before analysis to maintain mass tolerance of the instrument within low ppm limits. This acts as instrument level control on the mass tolerance. |
|  | Positive control: Pegfilgrastim reference standard. |
|  | Positive control for determination of oxidation level by LC-MS: Force-oxidized sample.  Positive control for N- and C-terminal analyses: Synthetic peptides. |
| Far UV CD | Positive control: Pegfilgrastim reference standard; Negative control: Formulation buffer and unrelated protein (rich in beta sheets) with secondary structure different than that of test sample |
| FTIR | Positive control: Pegfilgrastim reference standard; Negative control: Formulation buffer. |
| Near UV CD | Positive control: Pegfilgrastim reference standard; Negative control: formulation buffer and unrelated protein (BSA rich in alpha helix) with tertiary structure different than that of test sample |
| Intrinsic fluorescence | Positive control: Pegfilgrastim reference standard; Negative control: Formulation buffer and altered protein *i.e.,* protein denatured with guanidine HCl. |
| SPR | Positive control: Pegfilgrastim reference standard; Negative control: Formulation buffer and unrelated protein. |
| Cell based potency assay | Positive control: Pegfilgrastim reference standard; Negative control: No cell, no sample control. |
| AUC | Outsourced method performed using site SOP and calibration performed with standards |
| NMR | Outsourced analysis using site SOP and calibration performed with standards |
| DSC | Positive control: Pegfilgrastim reference standard; Negative control: Formulation buffer |
| SEC-MALS | Same as SE-HPLC |

MS, mass spectrometry; LC-MS, liquid chromatography MS; PEG, polyethylene glycol; CD, circular dichroism; FTIR, Fourier transform infrared spectroscopy; NMR, nuclear magnetic resonance; BSA, bovine serum albumin; SPR, surface plasmon resonance; SE-HPLC, size exclusion high performance liquid chromatography; AUC, analytical ultracentrifugation; SEC-MALS, size exclusion chromatography with multi-angle light scattering; CEX-HPLC, cation exchange HPLC; RP-HPLC, reverse phase HPLC; RP-UPLC-CAD, RP-UPLC with charged aerosol detection
