# Supplementary material for: Analytical sameness methodology for the evaluation of structural, physicochemical, and biological characteristics of Armlupeg: A pegfilgrastim biosimilar case study
Source: PLoS One. 2023 Aug 9;18(8):e0289745. doi: 10.1371/journal.pone.0289745 (PMC10411777; doi:10.1371/journal.pone.0289745)
Supplement: S3 Appendix — (DOCX) [file pone.0289745.s003.docx]

**S3 Appendix. Forced-degradation study results.**

## Oxidative/peroxide stress


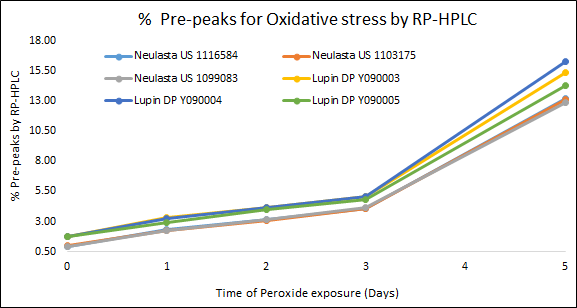


**Fig 1.** Graphical representation of % pre-peaks by RP-HPLC for oxidative stress.

Oxidative stress resulted in an increase in pre-peak impurities which was linear till day 3 and showed an exponential increase at day 5 for both the products.


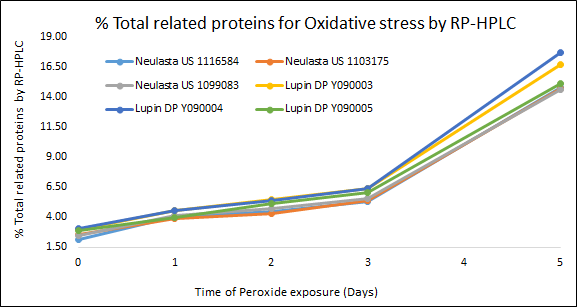


**Fig 2.** Graphical representation of % total related proteins by RP-HPLC for oxidative stress.

Oxidative stress resulted in an increase in total related proteins which was linear till day 3 and showed an exponential increase at day 5 for both the products.


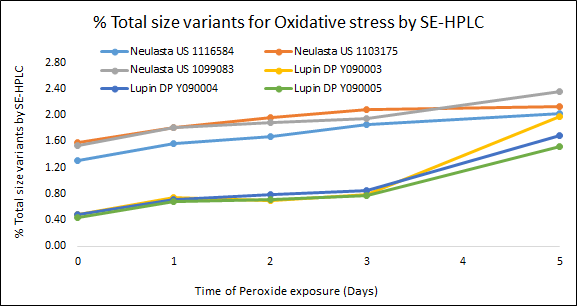


**Fig 3.** Graphical representation of % total size variants by SE-HPLC for oxidative stress.

Oxidative stress resulted in gradual increase in HMW species (aggregates). Aggregates were more in Neulasta® until day 4 but almost similar in both products on day 5.


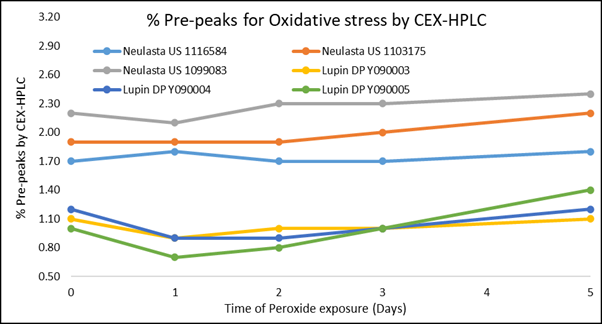


**Fig 4.** Graphical representation of % pre-peaks by CEX-HPLC for oxidative stress.

Oxidative stress resulted in no change in the pre-peak impurities in both the products.


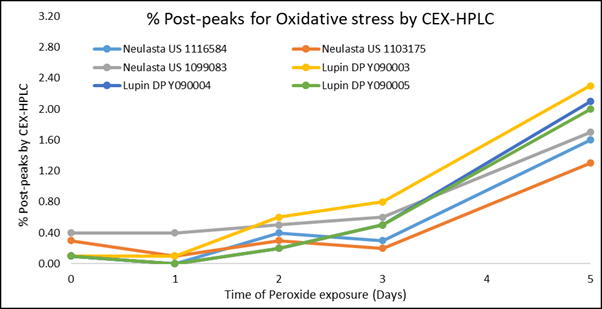


**Fig 5.** Graphical representation of % post-peaks by CEX-HPLC for oxidative stress.

Oxidative stress resulted in a linear increase in the post-peak impurities formation for both products.


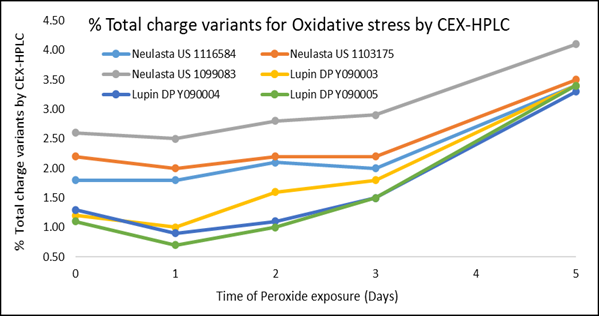


**Fig 6.** Graphical representation of % total charge variants by CEX-HPLC for oxidative stress.

Oxidative stress resulted in a linear increase in total charge variant formation for both products with comparable total charge variants at day 5.


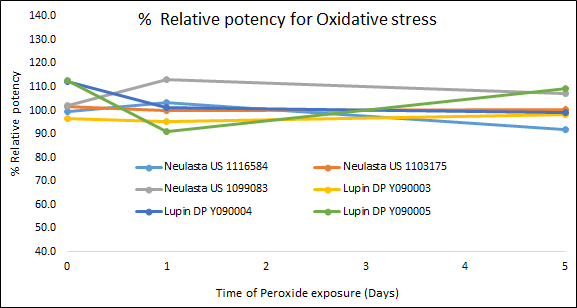


**Fig 7.** Graphical results of % relative potency for oxidative stress.

Oxidative stress had no impact on potency for both the products as assessed by the cell proliferation assay.

## pH-induced stress (acidic and basic)

### Exposure to acidic condition (pH 2.0)


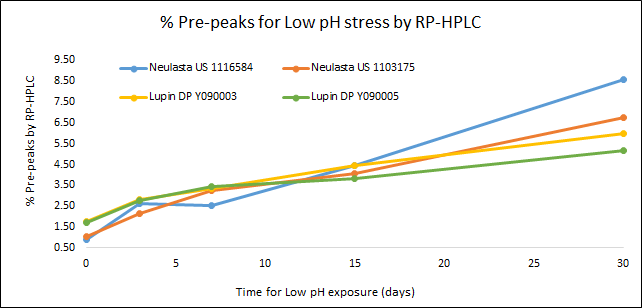


**Fig 8.** Graphical representation of % pre-peaks by RP-HPLC for low pH stress.

Low pH stress resulted in a linear increase in pre-peak impurities for both the products.


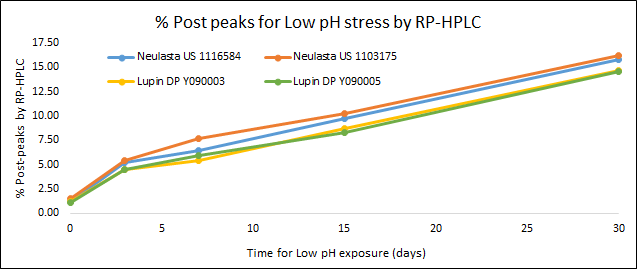


**Fig 9.** Graphical representation of % post-peaks by RP-HPLC for low pH stress.

Low pH stress resulted in a linear increase in post-peak impurities for both the products.

#
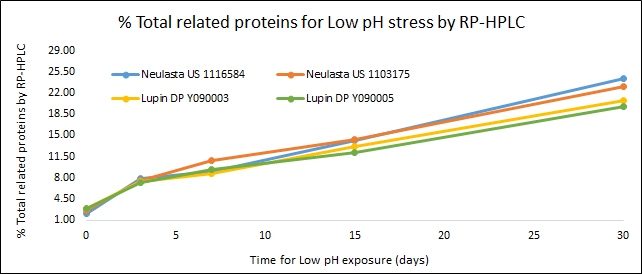


**Fig 10.** Graphical representation of % total related proteins by RP-HPLC for low pH stress.

Low pH stress resulted in a linear increase in total impurities for both the products.


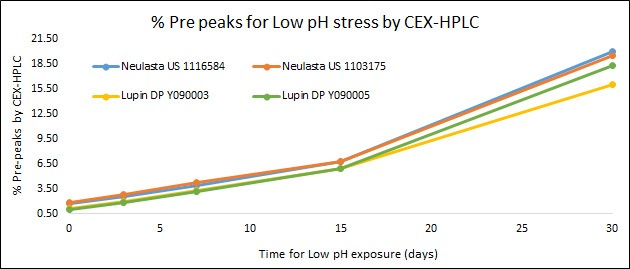


**Fig 11.** Graphical representation of % pre-peaks by CEX-HPLC for low pH stress.

Low pH stress resulted in a linear increase in the pre-peaks for both the products.

#
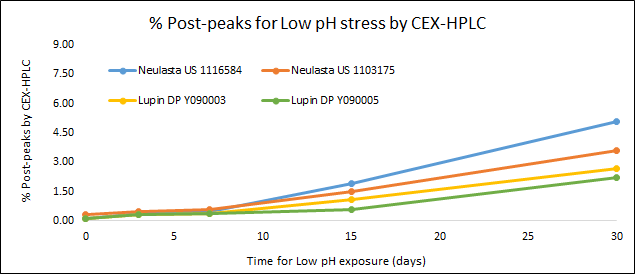


**Fig 12.** Graphical representation of % post-peaks by CEX-HPLC for low pH stress.

Low pH stress resulted in a linear increase in the post-peaks for both the products.


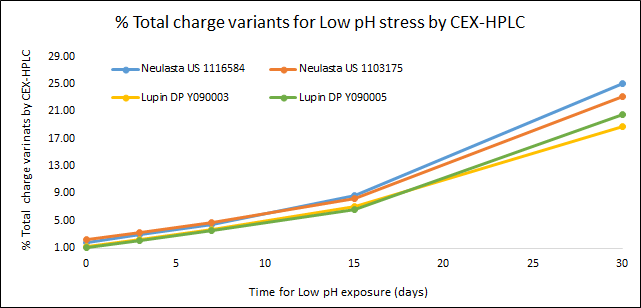


**Fig 13.** Graphical representation of % total charge variants by CEX-HPLC for low pH stress.

Low pH stress resulted in a linear increase in the total impurities for both the products.


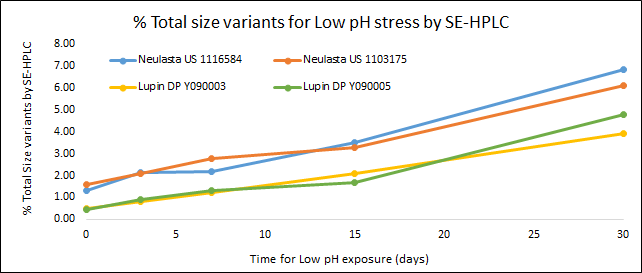


**Fig 14.** Graphical representation of % total size variants by SE-HPLC for low pH stress.

Low pH stress resulted in a linear increase in the aggregate formation for both the products.

**Table 1.** Free PEG content by RP-UPLC-CAD for Lupin’s Pegfilgrastim and Neulasta® after low pH stress for 30 days.

| Sample | Neulasta®  1116584 | Neulasta®  1103175 | Neulasta®  1099083 | Lupin’s Pegfilgrastim Y090003 | Lupin’s Pegfilgrastim Y090004 | Lupin’s Pegfilgrastim Y090005 |
| --- | --- | --- | --- | --- | --- | --- |
| Free PEG content (mg/mL) | 0.08 | 0.08 | 0.09 | 0.08 | 0.08 | 0.08 |

Low pH stress did not affect the free PEG content for both the products.


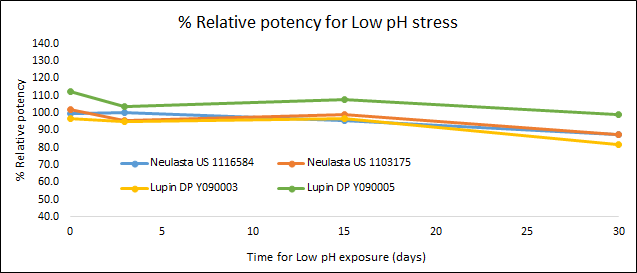


**Fig 15.** Graphical representation of % relative potency after low pH stress.

Low pH stress had no impact on potency for both the products as assessed by the cell proliferation assay.

### Exposure to basic condition (pH 9.0)


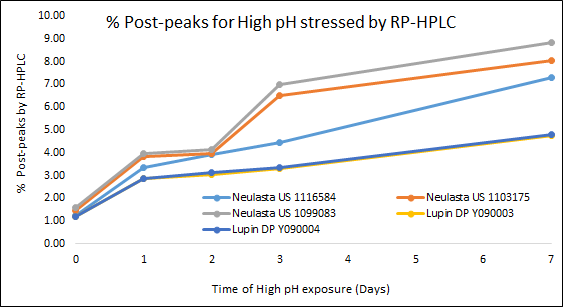


**Fig 16.** Graphical representation of % post-peaks by RP-HPLC for high pH stress.

High pH stress resulted in an increase in post-peak impurities which were lower in Lupin’s Pegfilgrastim.


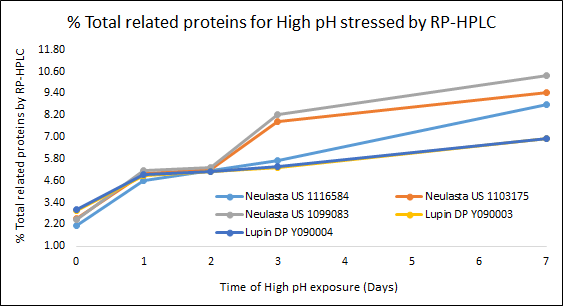


**Fig 17.** Graphical representation of % total related proteins by RP-HPLC for high pH stress.

High pH stress resulted in an increase in total impurities which were lower in Lupin’s Pegfilgrastim.


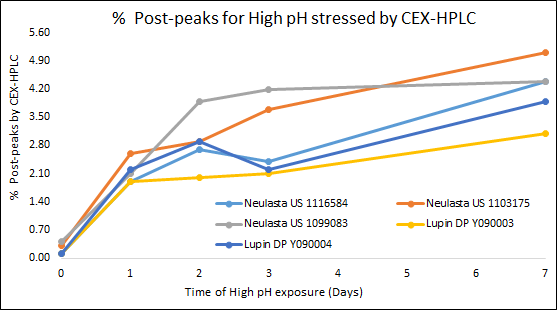


**Fig 18.** Graphical representation of % post-peaks by CEX-HPLC for high pH stress.

High pH stress resulted in an increase in post-peak impurities for both the products.


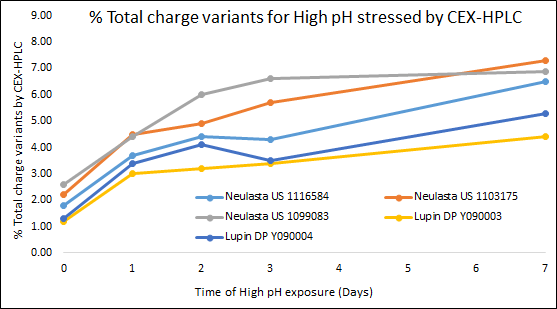


**Fig 19.** Graphical representation of % total charge variants by CEX-HPLC for high pH stress.

High pH stress resulted in an increase in total charge variants for both the products.


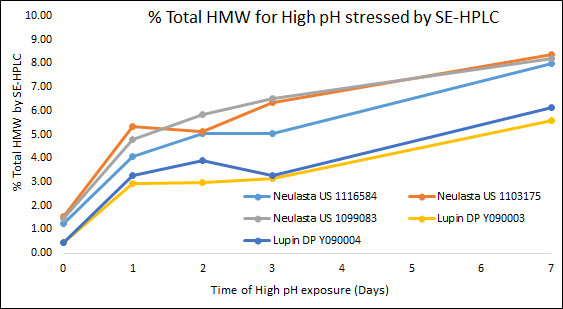


**Fig 20.** Graphical representation of % total HMW by SE-HPLC for high pH stress.

High pH stress resulted in an increase in total HMW for both the products.

**Table 2.** Free PEG content by RP-UPLC-CAD for Lupin’s Pegfilgrastim and Neulasta® after high pH stress for 7 days.

| Sample | Neulasta®  1116584 | Neulasta®  1103175 | Neulasta®  1099083 | Lupin’s Pegfilgrastim Y090003 | Lupin’s Pegfilgrastim Y090004 | Lupin’s Pegfilgrastim Y090005 |
| --- | --- | --- | --- | --- | --- | --- |
| Free PEG content (mg/mL) | 0.02 | 0.02 | 0.02 | 0.02 | 0.02 | 0.01 |

High pH stress resulted did not affect free PEG content for both the products.


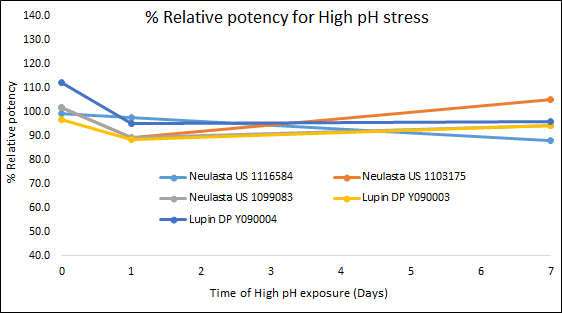


**Fig 21.** Graphical representation of % relative potency after high pH stress.

High pH stress had no impact on potency for both the products as assessed by the cell proliferation assay.

## Photolytic degradation (white light)
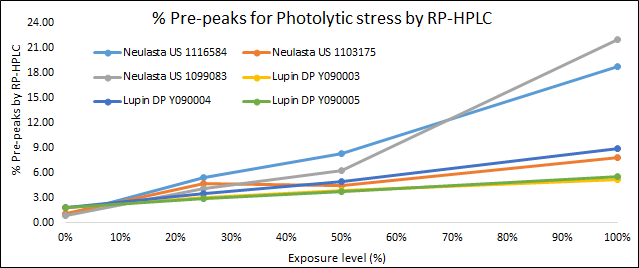


**Fig 22.** Graphical representation of % pre-peaks by RP-HPLC for photolytic stress.

Photolytic stress resulted in a linear increase in pre-peaks for both the products. The increase was more for Neulasta®.


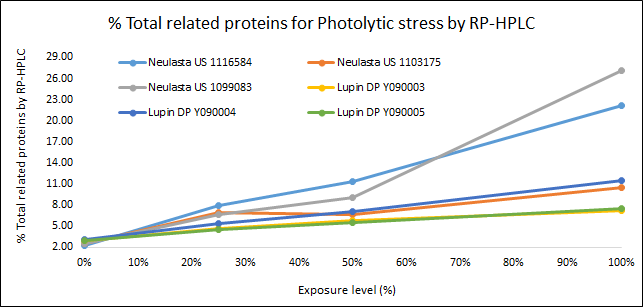


**Fig 23.** Graphical representation of % total related proteins by RP-HPLC for photolytic stress.

Photolytic stress resulted in a linear increase in total impurities for both the products. The increase was more for Neulasta®.


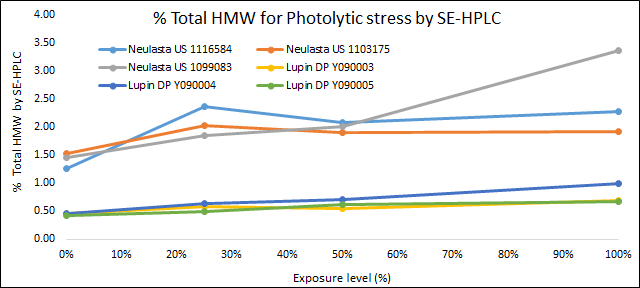


**Fig 24.** Graphical representation of % total HMW by SE-HPLC for photolytic stress.

Photolytic stress resulted in marginal increase in aggregates for both the products.


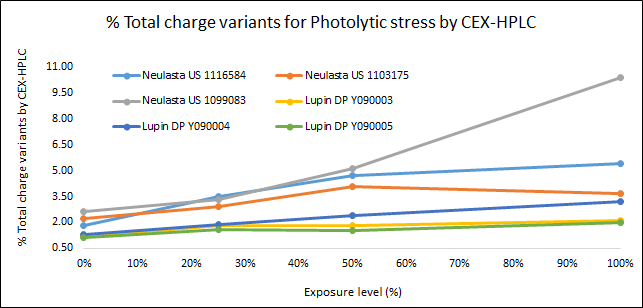


**Fig 25.** Graphical representation of % total charge variants by CEX-HPLC for photolytic stress.

Photolytic stress resulted in marginal increase in total charge variants for both the products. The increase was more for Neulasta®.

**Table 3.** Free PEG content by RP-UPLC-CAD for Lupin’s Pegfilgrastim and Neulasta® after 100% exposure to white light.

| Sample | Neulasta®  1116584 | Neulasta®  1103175 | Neulasta®  1099083 | Lupin’s Pegfilgrastim Y090003 | Lupin’s Pegfilgrastim Y090004 | Lupin’s Pegfilgrastim Y090005 |
| --- | --- | --- | --- | --- | --- | --- |
| Free PEG content (mg/mL) | 0.05 | 0.04 | 0.07 | 0.02 | 0.03 | 0.02 |

Photolytic stress resulted in a marginal increase in free PEG content for Neulasta®.

**Table 4.** % Relative potency of Neulasta® and Lupin’s Pegfilgrastim after 100% exposure to white light.

| **% Relative potency for photolytic (white light) stress** | | | | | | |
| --- | --- | --- | --- | --- | --- | --- |
| **Time point** | **Neulasta®** | | | **Lupin’s Pegfilgrastim** | | |
|  | **1116584** | **1103175** | **1099083** | **Y090003** | **Y090004** | **Y090005** |
| **Before exposure**  **(0 time point)** | 99.5 | 101.9 | 102 | 96.7 | 112.2 | 112.6 |
| **Photolytic Stress (100% exposure)** | 87.1 | 101.9 | 100.4 | 95.2 | 101.8 | 98.5 |

Photolytic stress had no impact on potency for both the products as assessed by the cell proliferation assay.

## Photolytic degradation (UV light)

**Table 5.** RP-HPLC results to evaluate degradation under UV stress.

| **Samples** | **Pre-peaks (%)** | | **Main peak (%)** | | **Post-peaks (%)** | | **Total related proteins (%)** | |
| --- | --- | --- | --- | --- | --- | --- | --- | --- |
|  | **Before UV exposure** | **UV exposed** | **Before UV exposure** | **UV exposed** | **Before UV exposure** | **UV exposed** | **Before UV exposure** | **UV exposed** |
| Neulasta® 1116584 | 0.91 | 54.20 | 97.83 | 41.35 | 1.25 | 4.45 | 2.16 | 58.65 |
| Neulasta® 1103175 | 1.05 | 48.33 | 97.48 | 47.13 | 1.48 | 4.56 | 2.53 | 52.89 |
| Neulasta® 1099083 | 0.90 | 54.27 | 97.51 | 39.66 | 1.59 | 6.08 | 2.48 | 60.35 |
| Lupin’s Pegfilgrastim Y090003 | 1.77 | 35.34 | 97.04 | 60.85 | 1.21 | 3.81 | 2.98 | 39.15 |
| Lupin’s Pegfilgrastim Y090004 | 1.80 | 41.12 | 96.98 | 55.18 | 1.21 | 3.71 | 3.01 | 44.82 |
| Lupin’s Pegfilgrastim Y090005 | 1.74 | 35.30 | 97.12 | 60.80 | 1.14 | 3.89 | 2.88 | 39.19 |

**Note:** Deamidation at Q108 position and dimer are included in the % post-peaks.

UV stress resulted in a significant increase in pre-peak impurities and total related proteins for both products.

**Table 6.** SE-HPLC results to evaluate degradation under UV stress.

| **Samples** | **Aggregate (%)** | | **HMW A+B (%)** | | **Main peak (%)** | | **Total size variants (%)** | |
| --- | --- | --- | --- | --- | --- | --- | --- | --- |
|  | **Before UV exposure** | **UV exposed** | **Before UV exposure** | **UV exposed** | **Before UV exposure** | **UV exposed** | **Before UV exposure** | **UV exposed** |
| Neulasta® 1116584 | 0.09 | 2.19 | 1.04 | 2.08 | 98.69 | 95.36 | 1.32 | 4.64 |
| Neulasta® 1103175 | 0.11 | 1.79 | 1.25 | 2.17 | 98.42 | 95.60 | 1.58 | 4.40 |
| Neulasta® 1099083 | 0.14 | 1.44 | 1.13 | 1.92 | 98.47 | 96.31 | 1.53 | 3.69 |
| Lupin’s Pegfilgrastim Y090003 | 0.09 | 1.57 | 0.34 | 1.08 | 99.52 | 97.22 | 0.49 | 2.79 |
| Lupin’s Pegfilgrastim Y090004 | 0.11 | 2.16 | 0.34 | 1.15 | 99.51 | 96.50 | 0.49 | 3.51 |
| Lupin’s PegfilgrastimY090005 | 0.10 | 1.58 | 0.30 | 1.12 | 99.56 | 97.09 | 0.45 | 2.92 |

UV stress resulted in a significant change in levels of aggregates and dimer, but not Des-PEG for both the products.

**Table 7.** CEX-HPLC results to evaluate degradation under UV stress.

| **Samples** | **Pre-peaks (%)** | | **Main peak (%)** | | **Post-peaks (%)** | | **Total charge variants (%)** | |
| --- | --- | --- | --- | --- | --- | --- | --- | --- |
|  | **Before UV exposure** | **UV exposed** | **Before UV exposure** | **UV exposed** | **Before UV exposure** | **UV exposed** | **Before UV exposure** | **UV exposed** |
| Neulasta® 1116584 | 1.7 | 14.6 | 98.2 | 80.5 | 0.1 | 4.9 | 1.8 | 19.5 |
| Neulasta® 1103175 | 1.9 | 12.4 | 97.8 | 83.5 | 0.3 | 4.1 | 2.2 | 16.50 |
| Neulasta® 1099083 | 2.2 | 15.8 | 97.4 | 81.4 | 0.4 | 2.7 | 2.6 | 18.4 |
| Lupin’s Pegfilgrastim Y090003 | 1.1 | 9.7 | 98.8 | 87.2 | 0.1 | 3.2 | 1.2 | 12.9 |
| Lupin’s Pegfilgrastim Y090004 | 1.2 | 12.1 | 98.7 | 84.5 | 0.1 | 3.4 | 1.3 | 15.5 |
| Lupin’s Pegfilgrastim Y090005 | 1.0 | 11.3 | 98.9 | 85.5 | 0.1 | 3.2 | 1.1 | 14.5 |

UV stress resulted in a significant increase in pre-peak, post-peak impurities, and total charged variants for both products. But pre-peak and post-peak impurities were lower in Lupin’s Pegfilgrastim.

**Table 8.** Free PEG content by RP-UHPLC-CAD for Lupin’s Pegfilgrastim and Neulasta® after exposure to UV stress.

| Sample | Neulasta®  1116584 | Neulasta®  1103175 | Neulasta®  1099083 | Lupin’s Pegfilgrastim Y090003 | Lupin’s Pegfilgrastim Y090004 | Lupin’s Pegfilgrastim Y090005 |
| --- | --- | --- | --- | --- | --- | --- |
| Free PEG content (mg/mL) | 0.09 | 0.04 | 0.03 | 0.03 | 0.03 | 0.01 |

UV stress had no impact on the free PEG content for both the products.

**Table 9.** % Relative potency of Neulasta® and Lupin’s Pegfilgrastim after 100% exposure to UV.

| **% Relative potency for photolytic stress (UV)** | | | | | | |
| --- | --- | --- | --- | --- | --- | --- |
| **Time point** | **Neulasta®** | | | **Lupin’s Pegfilgrastim** | | |
|  | **1116584** | **1103175** | **1099083** | **Y090003** | **Y090004** | **Y090005** |
| **Before exposure**  **(0 time point)** | 99.5 | 101.9 | 102 | 96.7 | 112.2 | 112.6 |
| **UV Stress**  **(100% exposure)** | 71.9 | 75.0 | 78.4 | 84.6 | 79.8 | 77.7 |

UV stress resulted in reduced functionality for both the products.

## Thermal stress


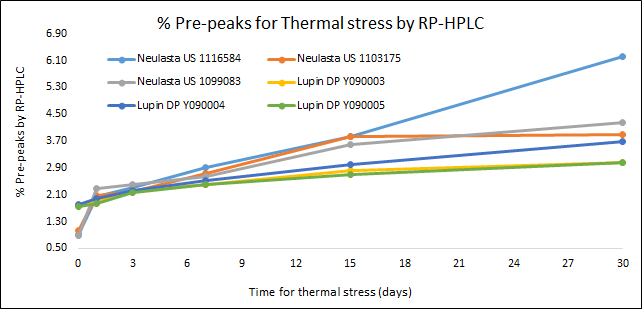


**Fig 26.** Graphical representation of % pre-peaks by RP-HPLC for thermal stress.

Thermal stress resulted in a gradual and linear increase in pre-peak impurities for both the products.


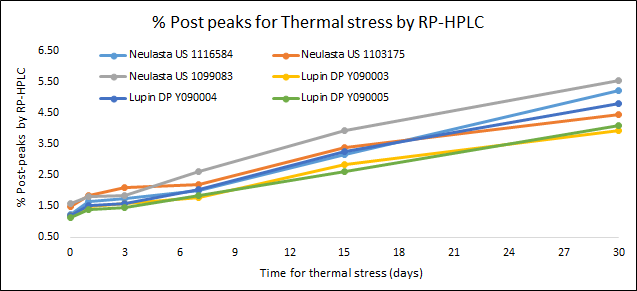


**Fig 27.** Graphical representation of % post-peaks by RP-HPLC for thermal stress.

Thermal stress resulted in a gradual and linear increase in post-peak impurities for both the products.


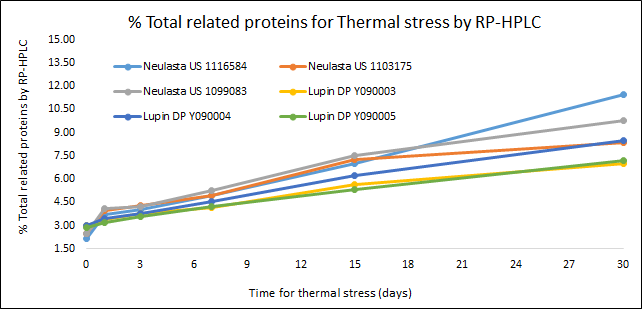


**Fig 28.** Graphical representation of % total related proteins by RP-HPLC for thermal stress.

Thermal stress resulted in a gradual and linear increase in total impurities for both the products.


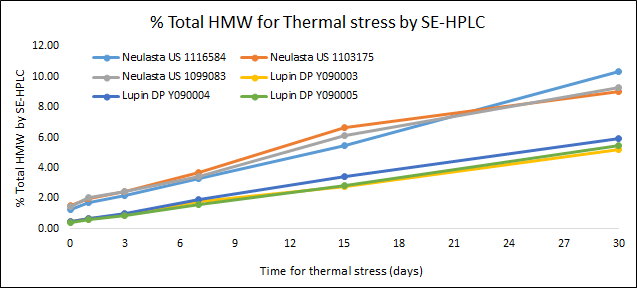


**Fig 29.** Graphical representation of % total HMW by SE-HPLC for thermal stress.

Thermal stress resulted in a linear increase in aggregates for both the products.


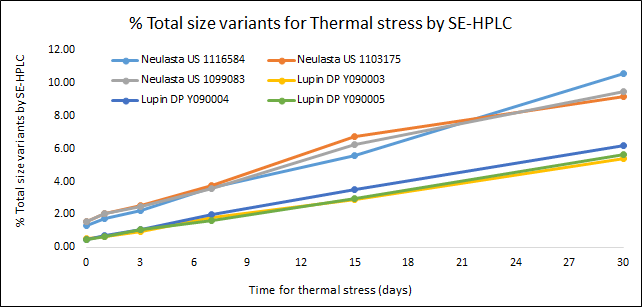


**Fig 30.** Graphical representation of % total size variants by SE-HPLC for thermal stress.

Thermal stress resulted in a linear increase in total size variants for both the products.


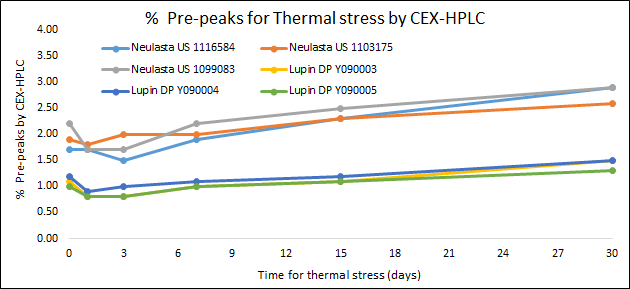


**Fig 31.** Graphical representation of % pre-peaks by CEX-HPLC for thermal stress.

Thermal stress resulted in a marginal increase in pre-peaks for both the products.


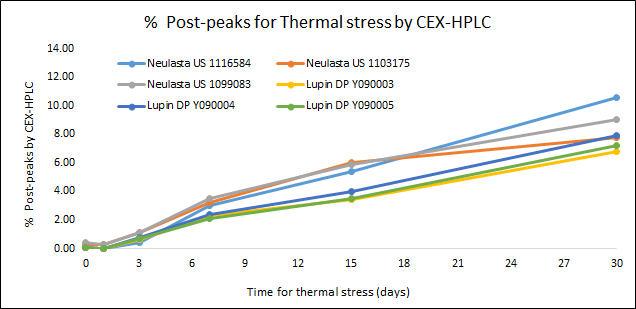


**Fig 32.** Graphical representation of % post-peaks by CEX-HPLC for thermal stress.

Thermal stress resulted in an increase in post-peaks for both the products.


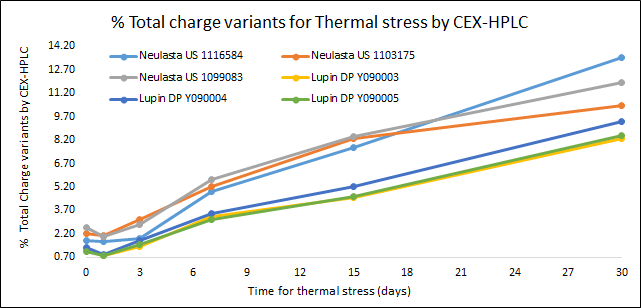


**Fig 33.** Graphical representation of % total charge variants by CEX-HPLC for thermal stress.

Thermal stress resulted in an increase in total charge variants for both the products.

**Table 10.** Free PEG content by RP-UHPLC-CAD for Lupin’s Pegfilgrastim and Neulasta® after thermal stress for 30 days.

| Sample | Neulasta® 1116584 | Neulasta® 1103175 | Neulasta® 1099083 | Lupin’s Pegfilgrastim Y090003 | Lupin’s Pegfilgrastim Y090004 | Lupin’s Pegfilgrastim Y090005 |
| --- | --- | --- | --- | --- | --- | --- |
| Free PEG content (mg/mL) | 0.48 | 0.40 | 0.42 | 0.33 | 0.40 | 0.37 |

Thermal stress results in a significant change in free PEG content for both the products.


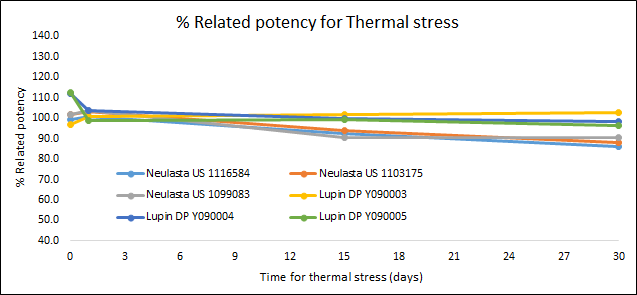


**Fig 34.** Graphical representation of % relative potency after thermal stress.

Thermal stress had no impact on the potency for both the products as assessed by the cell proliferation assay.

## Mechanical stress


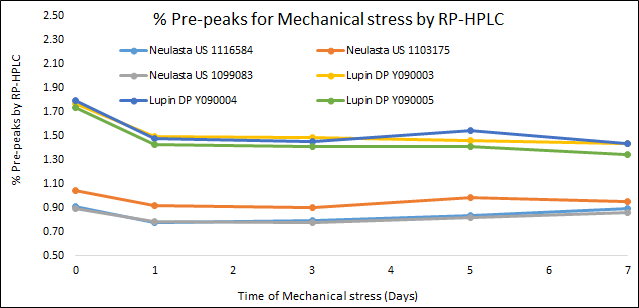


**Fig 35.** Graphical representation of % pre-peaks by RP-HPLC for mechanical stress.

Mechanical stress had no impact on the % of pre-peaks for both the products.


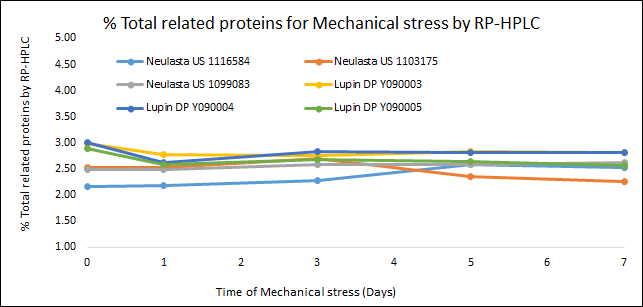


**Fig 36.** Graphical representation of % total related proteins by RP-HPLC for mechanical stress.

Mechanical stress had no impact on the % of total impurities for both the products.


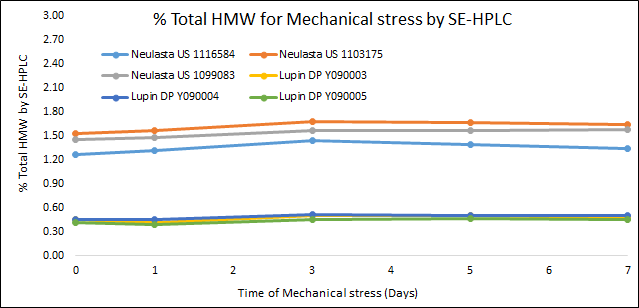


**Fig 37.** Graphical representation of % total HMW by SE-HPLC for mechanical stress.

Mechanical stress had no impact on the % of aggregates for both the products.


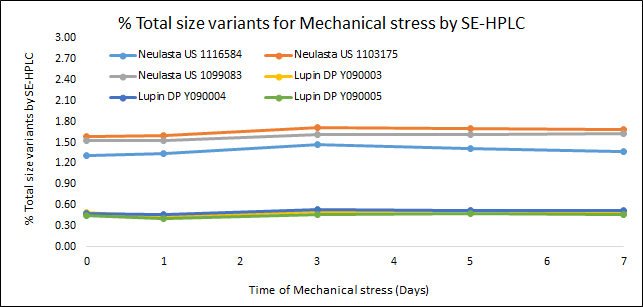


**Fig 38.** Graphical representation of % total size variants by SE-HPLC for mechanical stress.

Mechanical stress had no impact on the % of total size variants for both the products.


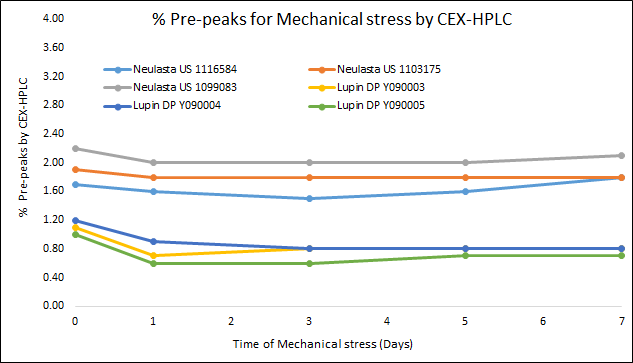


**Fig 39.** Graphical representation of % pre-peaks by CEX-HPLC for mechanical stress.

Mechanical stress had no impact on the % of pre-peaks for both the products.


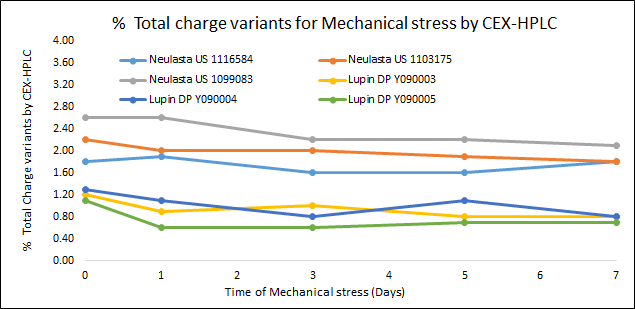


**Fig 40.** Graphical representation of % total charge variants by CEX-HPLC for mechanical stress.

Mechanical stress had no impact on the % of total charge variants for both the products.

**Table 11.** Free PEG content by RP-UHPLC-CAD for Lupin’s Pegfilgrastim and Neulasta® after mechanical stress for 7 days.

| Sample | Neulasta® 1116584 | Neulasta® 1103175 | Neulasta® 1099083 | Lupin’s Pegfilgrastim Y090003 | Lupin’s Pegfilgrastim Y090004 | Lupin’s Pegfilgrastim Y090005 |
| --- | --- | --- | --- | --- | --- | --- |
| Free PEG content (mg/mL) | 0.03 | 0.03 | 0.04 | 0.02 | 0.02 | 0.02 |

Mechanical stress had no impact on the free PEG content for both the products.


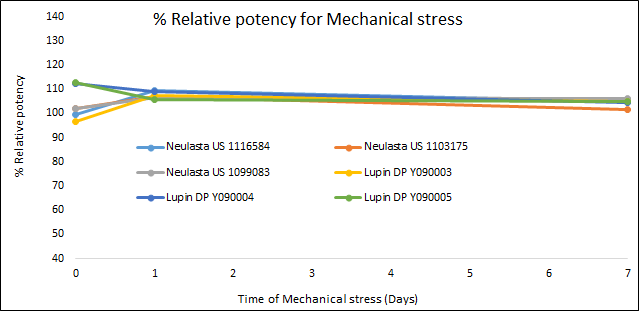


**Fig 41.** Graphical representation of % relative potency after mechanical stress.

Mechanical stress had no impact on the potency for the products as assessed by the cell proliferation assay.
