# Supplementary material for: Analytical sameness methodology for the evaluation of structural, physicochemical, and biological characteristics of Armlupeg: A pegfilgrastim biosimilar case study
Source: PLoS One. 2023 Aug 9;18(8):e0289745. doi: 10.1371/journal.pone.0289745 (PMC10411777; doi:10.1371/journal.pone.0289745)
Supplement: S4 Appendix — (DOCX) [file pone.0289745.s004.docx]

**S4 Appendix. Principal component analysis (PCA)**

For the critical quality attributes listed in S9 Table and S10 Table, PCA was performed with the reported values for each attribute in the multivariate mode using JMP version 16 (JMP performs standardization and normalization of data values).

| With all data values | After removal of outliers |
| --- | --- |
| 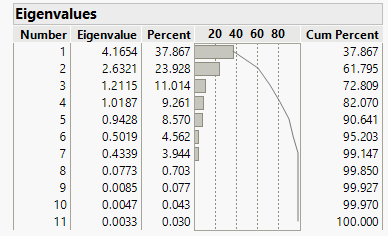 | 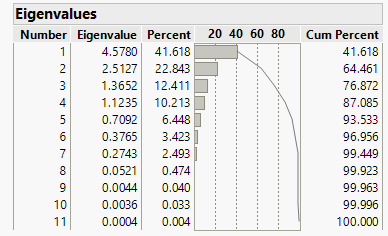 |
| 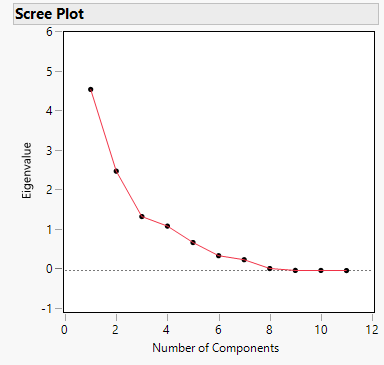 | 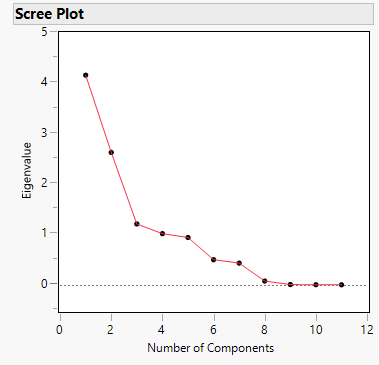 |

**Figure 1. Eigen values and scree plots for data with all values (left) and after removing outliers (right)**

Outliers were identified but not excluded from the data set because these outliers were also included for equivalence testing and/or quality range approach. Also, from the eigen values it was observed that in both cases the first four components explained >80 % of the variation in the data.


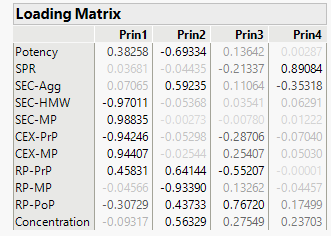


**Figure 2. Loading matrix.**

SEC, size exclusion high performance liquid chromatography; CEX, cation exchange HPLC; RP, reverse phase HPLC; Agg, , aggregates; HMW, high molecular weight species; MP, main peak, PrP, pre-peaks; PoP, post-peaks; Prin, Principal component; SPR, Surface plasmon resonance

In the loading matrix, principal component 1 (37.9 %) showed strong negative correlation with HMW observed by SE-HPLC and pre-peaks observed by CEX-HPLC and strong positive correlation with main peak observed by SE-HPLC and CEX-HPLC. It also showed weak positive correlation with potency and pre-peaks by RP-HPLC. Principal component 2 (23.9 %) showed negative correlation with potency and main peak by RP-HPLC and positive correlation with aggregates by SE-HPLC, pre-peaks by RP-HPLC, post-peaks by RP-HPLC, and protein concentration. Principal component 3 (11%) showed positive correlation with post-peaks by RP-HPLC. And principal component 4 (9.3%) showed positive correlation with binding kinetics by SPR.


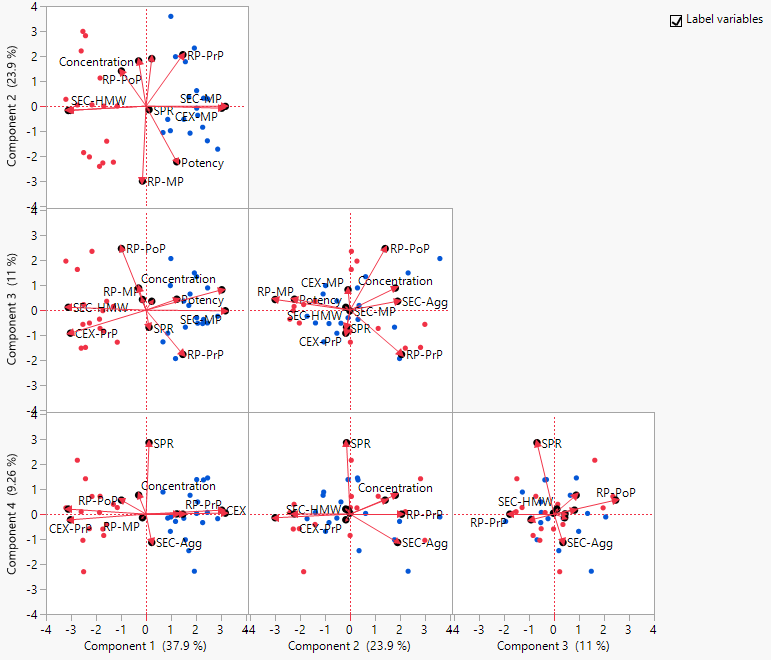

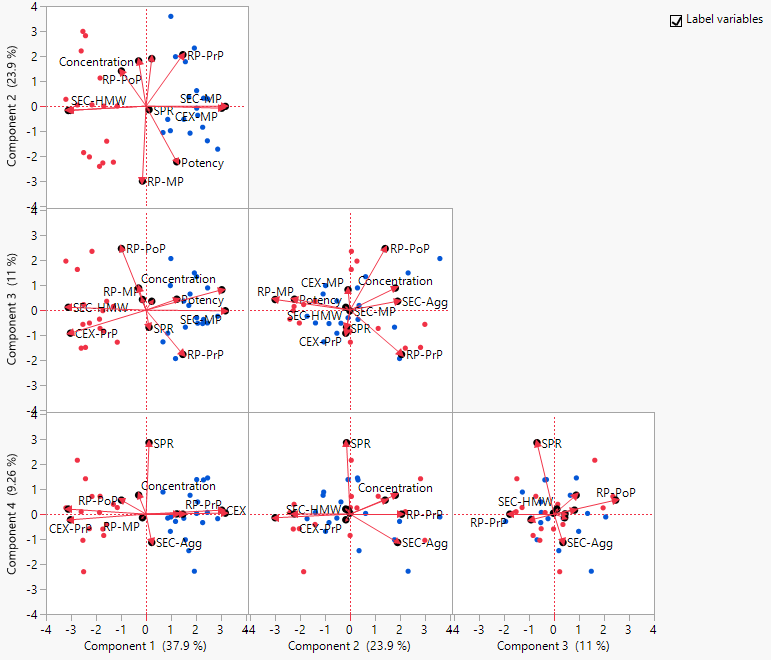


**Figure 3. Biplot graphs.** Red and blue dots denote scores of individual batches of Neulasta® and Lupin’s Pegfilgrastim respectively. The arrows denote each attribute in the loading matrix.

In the biplot graphs, Neulasta® and Lupin’s Pegfilgrastim scores cluster differently with principal component 1 versus principal components 2, 3, or 4. Principal component 1, which accounts for maximum variability, shows a strong correlation with the SE-HPLC and CEX-HPLC data. Neulasta® and Lupin’s Pegfilgrastim scores overlap for principal components 2, 3, and 4, which show a strong correlation with RP-HPLC, protein concentration, and potency. Lupin’s Pegfilgrastim had lower levels of dipegylated variants than Neulasta®. These dipegylated variants contribute to the HMW observed by SE-HPLC and pre-peaks observed by CEX-HPLC thus contributing to a difference in the overall purity, which was higher for Lupin’s Pegfilgrastim by these two methods. Overall, the conclusions by PCA match with the conclusions drawn from the individual attribute analysis.
