# Supplementary material for: Analytical sameness methodology for the evaluation of structural, physicochemical, and biological characteristics of Armlupeg: A pegfilgrastim biosimilar case study
Source: PLoS One. 2023 Aug 9;18(8):e0289745. doi: 10.1371/journal.pone.0289745 (PMC10411777; doi:10.1371/journal.pone.0289745)
Supplement: S1 Fig — (DOCX) [file pone.0289745.s005.docx]

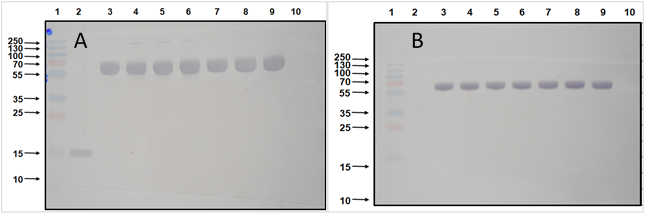


**S1 Fig.** **Western blot analysis** with (A) anti-filgrastim antibody and (B) anti-PEG antibody.

1-Marker; 2-Filgrastim IPRS; 3-Pegfilgrastim IPRS; 4-Neulasta® 1095928; 5-Neulasta® 1101290; 6-Neulasta® 1103175; 7-Lupin’s Pegfilgrastim V7100002; 8-Lupin’s Pegfilgrastim V7100006; 9-Lupin’s Pegfilgrastim V0100144; 10-Blank.

The anti-filgrastim and anti-PEG antibodies immunoreacted with both Lupin’s Pegfilgrastim and Neulasta®. The electrophoretic mobility of both the products was comparable.
