# Supplementary material for: Analytical sameness methodology for the evaluation of structural, physicochemical, and biological characteristics of Armlupeg: A pegfilgrastim biosimilar case study
Source: PLoS One. 2023 Aug 9;18(8):e0289745. doi: 10.1371/journal.pone.0289745 (PMC10411777; doi:10.1371/journal.pone.0289745)
Supplement: S2 Fig — (DOCX) [file pone.0289745.s006.docx]

###

**S2 Fig. FTIR spectra** (Representative images).

The second derivative FTIR spectra were similar for Neulasta® and Lupin’s Pegfilgrastim.
