# Supplementary material for: Analytical sameness methodology for the evaluation of structural, physicochemical, and biological characteristics of Armlupeg: A pegfilgrastim biosimilar case study
Source: PLoS One. 2023 Aug 9;18(8):e0289745. doi: 10.1371/journal.pone.0289745 (PMC10411777; doi:10.1371/journal.pone.0289745)
Supplement: S3 Fig — (DOCX) [file pone.0289745.s007.docx]

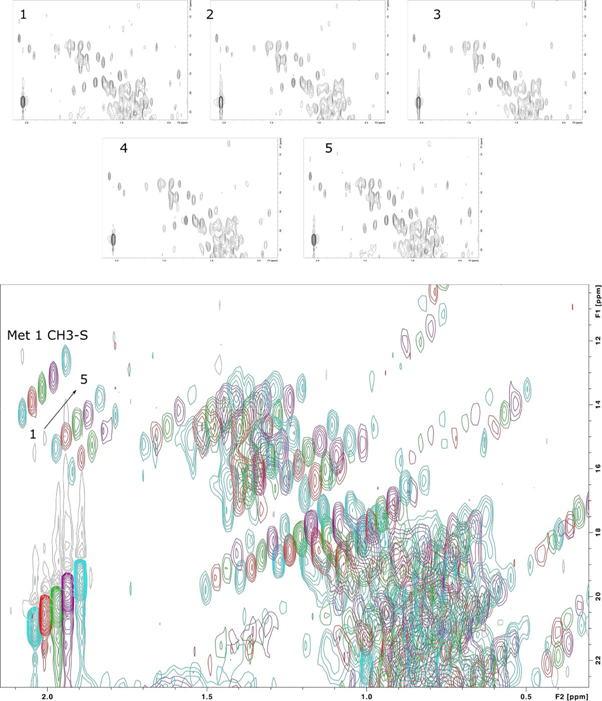


**S3 Fig. Fingerprint methyl/methylene regions from 2D ^1^H-^13^C HSQC NMR spectra at natural abundance of pegfilgrastim samples.** (1) Lupin’s Pegfilgrastim V0200039, (2) Lupin’s Pegfilgrastim V0200041, (3) Lupin’s Pegfilgrastim V0200043, (4) Neulasta® 1095928 and (5) Neulasta® 1116584. Bottom image: shifted overlay of the 5 spectra with the Met 1 methyl peak labelled. Cross peaks represent fingerprint C-Hn resonances from the polypeptide.

The NMR spectra for Neulasta® and Lupin’s Pegfilgrastim were comparable and overlapping.
