# Supplementary material for: Analytical sameness methodology for the evaluation of structural, physicochemical, and biological characteristics of Armlupeg: A pegfilgrastim biosimilar case study
Source: PLoS One. 2023 Aug 9;18(8):e0289745. doi: 10.1371/journal.pone.0289745 (PMC10411777; doi:10.1371/journal.pone.0289745)
Supplement: S4 Fig — (DOCX) [file pone.0289745.s008.docx]

**S4 Fig.** **Scatter plot of oxidation by LC-MS.** (A) Oxidation at M122, (B) Oxidation at M127, (C) Oxidation at M138, (D) Oxidation at M127, M138.

The type of oxidized species were similar between Neulasta® and Lupin’s Pegfilgrastim.
