# Supplementary material for: Analytical sameness methodology for the evaluation of structural, physicochemical, and biological characteristics of Armlupeg: A pegfilgrastim biosimilar case study
Source: PLoS One. 2023 Aug 9;18(8):e0289745. doi: 10.1371/journal.pone.0289745 (PMC10411777; doi:10.1371/journal.pone.0289745)
Supplement: S1 Table — (DOCX) [file pone.0289745.s009.docx]

**S1 Table. Comparison of theoretical mass and observed mass for corresponding peptide sequence of representative batches of Neulasta^®^ and Lupin’s Pegfilgrastim.**

| **Peptide Sequence** | | **Theoretical Mass (Da)** | **Neulasta® 1099084** | **Lupin’s Pegfilgrastim** | |
| --- | --- | --- | --- | --- | --- |
|  |  |  |  | **V9100102** | **Y900006** |
|  |  |  | **Observed Mass (Da)** | | |
| MTPLGPASSLPQSF | 1-14 | 1431.7068 | 1431.7116 | 1431.7076 | 1431.708 |
| LLKCLE | 15-20 | 774.4309 | 774.4312 | 774.4314 | 774.4317 |
| QVRKIQGDGAALQE | 21-34 | 1511.8056 | 1511.8067 | 1511.8071 | 1511.8070 |
| KLCATY | 35-40 | 754.3683 | 754.3689 | 754.3687 | 754.3692 |
| KLCHPEELVLL | 41-51 | 1349.7377 | 1349.7384 | 1349.7386 | 1349.7380 |
| GHSLGIPW | 52-59 | 865.4446 | 865.4451 | 865.4447 | 865.4447 |
| APLSSCPSQAL | 60-70 | 1129.5437 | 1129.5439 | 1129.5442 | 1129.5450 |
| QLAGCLSQLHSGLF | 71-84 | 1529.7660 | 1529.7707 | 1529.7701 | 1529.7656 |
| LYQGLLQALE | 85-94 | 1146.6285 | 1146.6297 | 1146.6311 | 1146.6297 |
| GISPE | 95-99 | 501.2435 | 501.2434 | 501.2436 | 501.2437 |
| LGPTLDTLQLD | 100-110 | 1184.6288 | 1184.6288 | 1184.6301 | 1184.6291 |
| VADFATTIWQQMEE | 111-124 | 1667.7501 | 1667.7503 | 1667.7519 | 1667.7479 |
| LGMAPALQPTQGAMPAFASAF | 125-145 | 2097.9991 | 2097.9922 | 2097.9922 | 2097.9896 |
| QRRAGGVL | 146-153 | 855.5039 | 855.5044 | 855.5035 | 855.5041 |
| VASHLQSFLE | 154-163 | 1129.5768 | 1129.5788 | 1129.5771 | 1129.5779 |
| VSYRVL | 164-169 | 735.4279 | 735.4283 | 735.4275 | 735.4280 |
| RHLAQP | 170-175 | 720.4031 | 720.4039 | 720.4039 | 720.4035 |

Reduced peptide mapping by MS analysis gave peptide sequences and fragment masses that were comparable for Neulasta® and Lupin’s Pegfilgrastim.
