# Supplementary material for: Analytical sameness methodology for the evaluation of structural, physicochemical, and biological characteristics of Armlupeg: A pegfilgrastim biosimilar case study
Source: PLoS One. 2023 Aug 9;18(8):e0289745. doi: 10.1371/journal.pone.0289745 (PMC10411777; doi:10.1371/journal.pone.0289745)
Supplement: S2 Table — (DOCX) [file pone.0289745.s010.docx]

**S2 Table. Comparison of the amino acid composition of Neulasta® and Lupin’s Pegfilgrastim.**

| **Amino acid composition (mole %)** | **Neulasta® (85 to 115% of mean)** | **Lupin’s Pegfilgrastim (Min-Max)** |
| --- | --- | --- |
| HIS | 2.64 to 3.58 | 2.87 to 3.20 |
| SER | 6.70 to 9.06 | 7.67 to 8.13 |
| ARG | 2.55 to 3.45 | 2.75 to 3.05 |
| GLY | 7.30 to 9.88 | 8.08 to 8.73 |
| ASP | 2.61 to 3.53 | 2.79 to 3.25 |
| GLX (E/Q) | 13.63 to 18.44 | 15.70 to 17.90 |
| THR | 3.84 to 5.20 | 4.17 to 4.58 |
| ALA | 9.43 to 12.76 | 11.02 to 11.79 |
| PRO | 6.78 to 9.17 | 7.71 to 8.17 |
| LYS | 2.05 to 2.78 | 2.37 to 2.53 |
| TYR | 1.58 to 2.13 | 1.63 to 1.94 |
| VAL | 3.71 to 5.02 | 3.88 to 4.37 |
| ILE | 2.04 to 2.76 | 2.16 to 2.42 |
| LEU | 16.99 to 22.98 | 19.49 to 20.09 |
| PHE | 3.16 to 4.27 | 3.43 to 3.94 |

All the amino acids quantified for Lupin’s Pegfilgrastim were within the generic acceptance range of 85 to 115% of the mean value for Neulasta®.
