# Supplementary material for: Analytical sameness methodology for the evaluation of structural, physicochemical, and biological characteristics of Armlupeg: A pegfilgrastim biosimilar case study
Source: PLoS One. 2023 Aug 9;18(8):e0289745. doi: 10.1371/journal.pone.0289745 (PMC10411777; doi:10.1371/journal.pone.0289745)
Supplement: S3 Table — (DOCX) [file pone.0289745.s011.docx]

**S3 Table. Comparison of the intact mass and polydispersity index of Neulasta® and Lupin’s Pegfilgrastim.**

| Sample | Batch Number | Intact Mass (Da) | Polydispersity Index |
| --- | --- | --- | --- |
| Neulasta® | 1074770 | 40503.8 | 1.0003 |
|  | 1099084 | 40524.1 | 1.0005 |
|  | 1095928 | 40503.2 | 1.0004 |
|  | 1099083 | 40459.1 | 1.0003 |
|  | 1103175 | 40458.3 | 1.0003 |
|  | 1116584 | 40480.6 | 1.0004 |
|  | 1101290 | 40529.9 | 1.0003 |
| Lupin’s Pegfilgrastim | V9100102 | 40503.5 | 1.0005 |
|  | V9100187 | 40504.8 | 1.0003 |
|  | V9100195 | 40522.9 | 1.0003 |
|  | V0200039 | 40611.0 | 1.0003 |
|  | V0200041 | 40503.7 | 1.0003 |
|  | V0200043 | 40456.4 | 1.0003 |
|  | V7100002 | 40586.9 | 1.0003 |
|  | V7100006 | 40634.1 | 1.0003 |
|  | V0100144 | 40482.1 | 1.0003 |

The mean intact mass and the mean polydispersity index were similar for Neulasta® and Lupin’s Pegfilgrastim.
