# Supplementary material for: Analytical sameness methodology for the evaluation of structural, physicochemical, and biological characteristics of Armlupeg: A pegfilgrastim biosimilar case study
Source: PLoS One. 2023 Aug 9;18(8):e0289745. doi: 10.1371/journal.pone.0289745 (PMC10411777; doi:10.1371/journal.pone.0289745)
Supplement: S4 Table — (DOCX) [file pone.0289745.s012.docx]

**S4 Table: Comparison of the intact mass and polydispersity index of released PEG-Met moiety from Neulasta® and Lupin’s Pegfilgrastim.**

| **Sample** | **Batch Number** | **Intact mass (Da)** | **Polydispersity Index** |
| --- | --- | --- | --- |
| Neulasta® | 1074770 | 22053.5 | 1.0006 |
|  | 1095928 | 21877.5 | 1.0007 |
|  | 1116584 | 22252.0 | 1.0006 |
| Lupin’s Pegfilgrastim | V0200039 | 22295.5 | 1.0007 |
|  | V0200041 | 22053.5 | 1.0007 |
|  | V0200043 | 21921.0 | 1.0007 |

The mean intact mass and the mean polydispersity index of the released PEG-Met moiety after one cycle of manual Edman degradation were similar for Neulasta® and Lupin’s Pegfilgrastim.
