# Supplementary material for: Analytical sameness methodology for the evaluation of structural, physicochemical, and biological characteristics of Armlupeg: A pegfilgrastim biosimilar case study
Source: PLoS One. 2023 Aug 9;18(8):e0289745. doi: 10.1371/journal.pone.0289745 (PMC10411777; doi:10.1371/journal.pone.0289745)
Supplement: S5 Table — (DOCX) [file pone.0289745.s013.docx]

**S5 Table. Comparison of theoretical masses for des-PEG-Met filgrastim and other PTC adducts.**

| **Protein molecule** | **Theoretical  mass (Da)** | **Neulasta®** | | | **Lupin’s Pegfilgrastim** | | |
| --- | --- | --- | --- | --- | --- | --- | --- |
|  |  | **1074770** | **1095928** | **1116584** | **V0200039** | **V0200041** | **V0200043** |
|  |  | **Observed mass (Da)** | | | | | |
| Filgrastim | 18799 | ND | ND | ND | ND | ND | ND |
| des-PEG-Met Filgrastim | 18667 | 18667 | 18667 | 18667 | 18667 | 18667 | 18667 |
| des-PEG-Met Filgrastim + 1 PTC | 18803 | 18803 | 18802 | 18802 | 18803 | 18802 | 18803 |
| des-PEG-Met Filgrastim + 2 PTC | 18938 | 18938 | 18937 | 18938 | 18938 | 18938 | 18938 |
| des-PEG-Met Filgrastim + 3 PTC | 19073 | 19073 | 19073 | 19073 | 19073 | 19073 | 19073 |
| des-PEG-Met Filgrastim + 4 PTC | 19208 | 19208 | 19208 | 19208 | 19208 | 19208 | 19208 |

PTC, phenylthiocarbamyl

The masses for des-PEG-Met filgrastim and other respective phenylthiocarbamyl adducts were similar across all tested batches of Neulasta® and Lupin’s Pegfilgrastim.
