# Supplementary material for: Analytical sameness methodology for the evaluation of structural, physicochemical, and biological characteristics of Armlupeg: A pegfilgrastim biosimilar case study
Source: PLoS One. 2023 Aug 9;18(8):e0289745. doi: 10.1371/journal.pone.0289745 (PMC10411777; doi:10.1371/journal.pone.0289745)
Supplement: S6 Table — (DOCX) [file pone.0289745.s014.docx]

**S6 Table. Comparison of theoretical mass and observed mass for corresponding peptide sequence after one cycle of Edman degradation of representative batches of Neulasta^®^ and Lupin’s Pegfilgrastim.**

| **Peptide Sequence** | **Amino acid residue no.** | **Theoretical Mass (Da)** | **Neulasta®** | | | **Lupin’s Pegfilgrastim** | | |
| --- | --- | --- | --- | --- | --- | --- | --- | --- |
|  |  |  | **1074770** | **1095928** | **1116584** | **V0200039** | **V0200041** | **V0200043** |
| MTPLGPASSLPQSFLLKCLE | 1-20 | 2189.11 | ND | ND | ND | ND | ND | ND |
| TPLGPASSLPQSFLLKCLE | 2-20 | 2058.07 | 2058.07 | 2058.07 | 2058.07 | 2058.07 | 2058.07 | 2058.07 |
| QVRKIQGD | 21-28 | 942.52 | 942.52 | 942.52 | 942.52 | 942.52 | 942.52 | 942.52 |
| GAALQE | 29-34 | 587.29 | 587.29 | 587.29 | 587.29 | 587.29 | 587.29 | 587.29 |
| KLCATYKLCHPE | 35-46 | 1520.70 | 1520.71 | 1520.71 | 1520.71 | 1520.71 | 1520.71 | 1520.71 |
| KLCATYKLCHPEE | 35-47 | 1649.74 | 1649.74 | 1649.74 | 1649.74 | 1649.74 | 1649.74 | 1649.74 |
| LVLLGHSLGIPWAPLSSCPSQALQLAGCLSQLHSGLFLYQGLLQALE | 48-94 | 5057.64 | 5057.61 | 5057.65 | 5057.65 | 5057.65 | 5057.65 | 5057.65 |
| GISPE | 95-99 | 501.24 | 501.24 | 501.24 | 501.24 | 501.24 | 501.24 | 501.24 |
| LGPTLD | 100-105 | 614.33 | 614.33 | 614.33 | 614.33 | 614.33 | 614.33 | 614.33 |
| TLQLD | 106-110 | 588.31 | 588.31 | 588.31 | 588.31 | 588.31 | 588.31 | 588.31 |
| TLQLDVAD | 106-113 | 873.44 | 873.44 | 873.44 | 873.44 | 873.44 | 873.44 | 873.44 |
| FATTIWQQMEE | 114-124 | 1382.62 | 1382.62 | 1382.62 | 1382.62 | 1382.62 | 1382.62 | 1382.62 |
| LGMAPALQPTQGAMPAFASAFQRRAGGVLVASHLQSFLE | 125-163 | 4025.08 | 4025.09 | 4025.09 | 4025.09 | 4025.05 | 4025.09 | 4025.09 |
| VSYRVLRHLAQP | 164-175 | 1437.82 | 1437.82 | 1437.82 | 1437.82 | 1437.82 | 1437.85 | 1437.82 |

Peptide mapping post one cycle of Edman degradation showed similar peptide fragment masses between Neulasta® and Lupin’s Pegfilgrastim.
