# Supplementary material for: Analytical sameness methodology for the evaluation of structural, physicochemical, and biological characteristics of Armlupeg: A pegfilgrastim biosimilar case study
Source: PLoS One. 2023 Aug 9;18(8):e0289745. doi: 10.1371/journal.pone.0289745 (PMC10411777; doi:10.1371/journal.pone.0289745)
Supplement: S7 Table — (DOCX) [file pone.0289745.s015.docx]

**S7 Table. Comparison of observed mass of N- and C-terminal variants by LC-MS.**

| **Sample** |  | **N-terminal Formyl (f-Met)** | **N-terminal** | **N-1** | **N-2** | **C-terminal** | **C-1** | **C-2** |
| --- | --- | --- | --- | --- | --- | --- | --- | --- |
|  | **Theoretical m/z** | **1109.0689** | **1095.0714** | **1029.5512** | **979.0273** | **480.2813** | **671.3917** | **607.3624** |
|  | **Charge state (z)** | **2** | **2** | **2** | **2** | **3** | **2** | **2** |
|  | **Batch Number** | **Observed m/z** | | | | | | |
| Neulasta® | 1074770 | ND | 1095.0721 | 1029.5561 | ND | 480.2815 | ND | ND |
|  | 1099084 | ND | 1095.0719 | 1029.5554 | ND | 480.2815 | ND | ND |
|  | 1095928 | ND | 1095.0709 | 1029.5556 | ND | 480.2813 | ND | ND |
|  | 1099083 | ND | 1095.0704 | 1029.5544 | ND | 480.2808 | ND | ND |
|  | 1103175 | ND | 1095.0704 | 1029.5542 | ND | 480.2811 | ND | ND |
|  | 1116584 | ND | 1095.0705 | 1029.5540 | ND | 480.2809 | ND | ND |
|  | 1101290 | ND | 1095.0708 | 1029.5498 | ND | 480.2810 | ND | ND |
| Lupin’s Pegfilgrastim | V9100102 | ND | 1095.0703 | 1029.5528 | ND | 480.2810 | ND | ND |
|  | V9100187 | ND | 1095.0719 | 1029.5554 | ND | 480.2814 | ND | ND |
|  | V9100195 | ND | 1095.0723 | 1029.5555 | ND | 480.2816 | ND | ND |
|  | V0200039 | ND | 1095.0760 | 1029.5547 | ND | 480.2816 | ND | ND |
|  | V0200041 | ND | 1095.0722 | 1029.5562 | ND | 480.2818 | ND | ND |
|  | V0200043 | ND | 1095.0723 | ND | ND | 480.2818 | ND | ND |
|  | V7100002 | ND | 1095.0712 | 1029.5505 | ND | 480.2813 | ND | ND |
|  | V7100006 | ND | 1095.0706 | 1029.5505 | ND | 480.2808 | ND | ND |
|  | V8100017 | ND | 1095.0707 | 1029.5499 | ND | 480.2810 | ND | ND |
|  | V8100046 | ND | 1095.0710 | 1029.5501 | ND | 480.2814 | ND | ND |
|  | V0100144 | ND | 1095.0712 | ND | ND | 480.2809 | ND | ND |

ND, not determined

The type of N- and C-terminal variants were same in Neulasta® and Lupin’s Pegfilgrastim.
