# Supplementary material for: Analytical sameness methodology for the evaluation of structural, physicochemical, and biological characteristics of Armlupeg: A pegfilgrastim biosimilar case study
Source: PLoS One. 2023 Aug 9;18(8):e0289745. doi: 10.1371/journal.pone.0289745 (PMC10411777; doi:10.1371/journal.pone.0289745)
Supplement: S8 Table — (DOCX) [file pone.0289745.s016.docx]

# **S8 Table. Molar extinction coefficient determination (Edelhoch).**

| **Sample** | **Batch Number** | **Molar Extinction Coefficient** |
| --- | --- | --- |
| Neulasta® | 1074770 | 0.84 |
|  | 1099084 | 0.85 |
|  | 1116584 | 0.85 |
|  | 1095928 | 0.84 |
|  | 1101290 | 0.85 |
|  | 1103175 | 0.83 |
| Lupin’s Pegfilgrastim | V9100102 | 0.82 |
|  | V0200039 | 0.82 |
|  | V0200043 | 0.84 |
|  | Y790003 | 0.85 |
|  | Y790005 | 0.86 |
|  | Y900006 | 0.84 |
|  | V7100002 | 0.84 |
|  | V7100006 | 0.85 |
|  | V0100144 | 0.83 |

The extinction coefficient determined by Edelhoch method was similar for Neulasta® and Lupin’s Pegfilgrastim.
