# Supplementary material for: Analytical sameness methodology for the evaluation of structural, physicochemical, and biological characteristics of Armlupeg: A pegfilgrastim biosimilar case study
Source: PLoS One. 2023 Aug 9;18(8):e0289745. doi: 10.1371/journal.pone.0289745 (PMC10411777; doi:10.1371/journal.pone.0289745)
Supplement: S9 Table — (DOCX) [file pone.0289745.s017.docx]

**S9 Table. Descriptive statistics summary.**

| **Functional attribute** | **Product** | **n** | **Mean (%)** | **95% CI of mean (%)** | | **SD** |
| --- | --- | --- | --- | --- | --- | --- |
|  |  |  |  | **Lower** | **Upper** |  |
| Relative potency by cell proliferation assay | Neulasta® | 12 | 99.4 | 93.6 | 105.2 | 9.147 |
|  | Lupin’s Pegfilgrastim | 18 | 103.5 | 99.8 | 107.3 | 7.577 |
| Binding kinetics with Filgrastim receptor using SPR | Neulasta® | 12 | 9.01E-11 | 7.69E-11 | 1.03E-10 | 2.07E-11 |
|  | Lupin’s Pegfilgrastim | 18 | 9.47E-11 | 8.48E-11 | 1.05E-10 | 1.98E-11 |
| Aggregate by  SE-HPLC | Neulasta® | 12 | 0.14 | 0.13 | 0.16 | 0.03 |
|  | Lupin’s Pegfilgrastim | 18 | 0.14 | 0.12 | 0.17 | 0.05 |
| HMW A + B by  SE-HPLC | Neulasta® | 12 | 1.13 | 1.08 | 1.17 | 0.07 |
|  | Lupin’s Pegfilgrastim | 18 | 0.37 | 0.32 | 0.42 | 0.10 |
| Main peak by  SE-HPLC | Neulasta® | 12 | 98.52 | 98.46 | 98.59 | 0.10 |
|  | Lupin’s Pegfilgrastim | 18 | 99.43 | 99.35 | 99.50 | 0.15 |
| Pre-peaks by  CEX-HPLC | Neulasta® | 12 | 2.0 | 1.9 | 2.1 | 0.14 |
|  | Lupin’s Pegfilgrastim | 18 | 1.2 | 1.0 | 1.3 | 0.22 |
| Main peak by  CEX-HPLC | Neulasta® | 12 | 97.9 | 97.8 | 98.0 | 0.20 |
|  | Lupin’s Pegfilgrastim | 18 | 98.8 | 98.7 | 98.9 | 0.24 |
| Pre-peaks by  RP-HPLC | Neulasta® | 12 | 1.48 | 1.17 | 1.78 | 0.48 |
|  | Lupin’s Pegfilgrastim | 18 | 1.76 | 1.59 | 1.92 | 0.34 |
| Main peak by  RP-HPLC | Neulasta® | 12 | 97.04 | 96.70 | 97.39 | 0.54 |
|  | Lupin’s Pegfilgrastim | 18 | 96.93 | 96.74 | 97.12 | 0.39 |
| Post peaks by  RP-HPLC | Neulasta® | 12 | 1.06 | 1.02 | 1.10 | 0.06 |
|  | Lupin’s Pegfilgrastim | 18 | 1.07 | 1.00 | 1.14 | 0.15 |
| Deamidated by  RP-HPLC | Neulasta® | 12 | 0.41 | 0.37 | 0.45 | 0.06 |
|  | Lupin’s Pegfilgrastim | 18 | 0.26 | 0.22 | 0.30 | 0.07 |
| Protein content | Neulasta® | 12 | 10.4 | 10.2 | 10.5 | 0.24 |
|  | Lupin’s Pegfilgrastim | 18 | 10.4 | 10.3 | 10.5 | 0.26 |

n, number of batches tested; CI, confidence interval; SD, standard deviation; CEX-HPLC, cation exchange high performance liquid chromatography; RP-HPLC, reverse phase HPLC

Lupin’s Pegfilgrastim was similar to Neulasta® as demonstrated by equivalence testing, or a 1-sided or 2-sided quality range approach for analysis.
