# Supplementary material for: Analytical sameness methodology for the evaluation of structural, physicochemical, and biological characteristics of Armlupeg: A pegfilgrastim biosimilar case study
Source: PLoS One. 2023 Aug 9;18(8):e0289745. doi: 10.1371/journal.pone.0289745 (PMC10411777; doi:10.1371/journal.pone.0289745)
Supplement: S10 Table — (DOCX) [file pone.0289745.s018.docx]

**S10 Table. Brief summary of analytical assessment for ‘high to very high’ risk scoring attributes.**

| **Functional attribute** | **Difference of means** | **95% CI of difference of means** | **Equivalence margin** |
| --- | --- | --- | --- |
| Relative potency by cell proliferation assay | 4.1% | -1.12, 9.32 | -18.3, 18.3 |
| Binding kinetics with filgrastim receptor using SPR | 4.64E-12 | -8.16E-12, 1.75E-11 | -4.14E-11, 4.14E-11 |
| Protein content | -0.003 | -0.16, 0.16 | -0.5, 0.5 |

CI, confidence interval; SPR, surface plasmon resonance

The 95 % CI of difference of means is within the equivalence margin derived from Neulasta® values demonstrating equivalence of Lupin’s Pegfilgrastim with Neulasta® for the three attributes.
